# One stone, three birds: one AIEgen with three colors for fast differentiation of three pathogens†

**DOI:** 10.1039/d0sc00256a

**Published:** 2020-04-14

**Authors:** Chengcheng Zhou, Meijuan Jiang, Jian Du, Haotian Bai, Guogang Shan, Ryan T. K. Kwok, Joe H. C. Chau, Jun Zhang, Jacky W. Y. Lam, Peng Huang, Ben Zhong Tang

**Affiliations:** Department of Chemistry, Hong Kong Branch of Chinese National Engineering Research Center for Tissue Restoration and Reconstruction, Department of Chemical and Biological Engineering, The Hong Kong University of Science and Technology Clear Water Bay Kowloon Hong Kong China tangbenz@ust.hk; Guangdong Key Laboratory for Biomedical Measurements and Ultrasound Imaging, Laboratory of Evolutionary Theranostics, School of Biomedical Engineering, Health Science Center, Shenzhen University Shenzhen 518060 China; Key Laboratory of Optoelectronic Devices and Systems of Ministry of Education and Guangdong Province, College of Optoelectronic Engineering, Shenzhen University Shenzhen 518060 China; HKUST Shenzhen Research Institute No. 9 Yuexing 1st RD, South Area, Hi-tech Park Nanshan Shenzhen 518057 China; Urinary Surgery, The First Affiliated Hospital of Soochow University Pinghai Road Suzhou 215006 China; State Key Laboratory of Luminescent Materials and Devices, Center for Aggregation-Induced Emission, Guangzhou International Campus, South China University of Technology Guangzhou 510640 China

## Abstract

Visually identifying pathogens favors rapid diagnosis at the point-of-care testing level. Here, we developed a microenvironment-sensitive aggregation-induced emission luminogen (AIEgen), namely IQ–Cm, for achieving fast discrimination of Gram-negative bacteria, Gram-positive bacteria and fungi by the naked-eye. With a twisted donor–acceptor and multi-rotor structure, IQ–Cm shows twisted intramolecular charge transfer (TICT) and AIE properties with sensitive fluorescence color response to the microenvironment of pathogens. Driven by the intrinsic structural differences of pathogens, IQ–Cm with a cationic isoquinolinium moiety and a membrane-active coumarin unit as the targeting and interacting groups selectively locates in different sites of three pathogens and gives three naked-eye discernible emission colors. Gram-negative bacteria are weak pink, Gram-positive bacteria are orange-red and fungi are bright yellow. Therefore, based on their distinctive fluorescence response, IQ–Cm can directly discriminate the three pathogens at the cell level under a fluorescence microscope. Furthermore, we demonstrated the feasibility of IQ–Cm as a visual probe for fast diagnosis of urinary tract infections, timely monitoring of hospital-acquired infection processes and fast detection of molds in the food field. This simple visualization strategy based on one single AIEgen provides a promising platform for rapid pathogen detection and point-of-care diagnosis.

## Abstract 2

A simple AIEgen with three emission colors achieves rapid identification of Gram-negative bacteria, Gram-positive bacteria and fungi.

## Graphical abstract

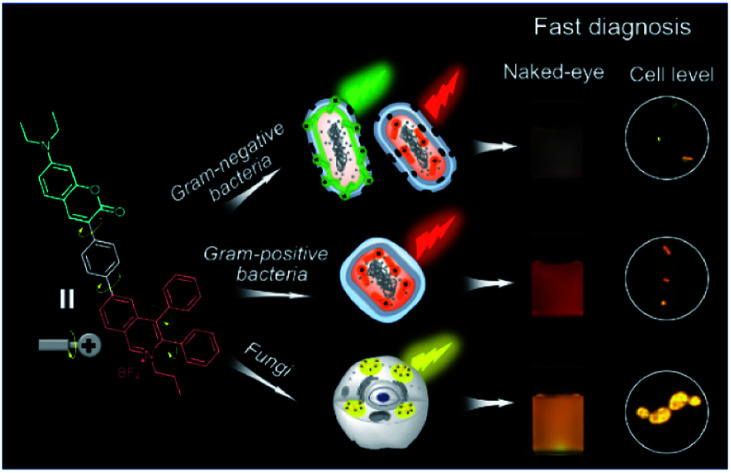

## Introduction

Pathogenic bacteria and fungi are everywhere and pose severe threats to human health and safety.^1–3^ Their infections cause many severe diseases, such as urinary tract infections (UTIs), sepsis and pneumonia.^4,5^ The fast identification of pathogen type is the first and most critical step to reduce the abuse of antibiotics and ensure effective treatment.^2,6^ The gold standard of diagnosis, pathogen culturing, generally takes several days and results in delayed reports.^3,7^ The Gram-staining method can overcome such time limitations by enabling direct observation of the colors of pathogens after staining, but its accuracy rate is low (about 40–60%) due to its complicated multi-step procedure and the low sensitivity of colorimetry.^8–10^ Also, the method can't effectively discriminate between Gram-positive (G+) bacteria and fungi because they both present blue/purple color. Without timely and reliable pathogen information, inadequate antimicrobial therapy could be performed.^11^ For instance, in clinics, empirical or broad-spectrum antibiotic therapy is often employed for first-hand UTI treatment.^12,13^ This not only leads to compromised treatment and high chances of hospital-acquired infection, but also significantly promotes the emergence of drug-resistant pathogens.^14^ Therefore, a simple and reliable visualization strategy is urgently needed for fast discrimination of pathogens.^3,6,15,16^ In particular, achieving direct naked-eye visual identification of pathogens will be very beneficial for rapid diagnosis at the point-of-care testing level.^17^

**Scheme 1:**
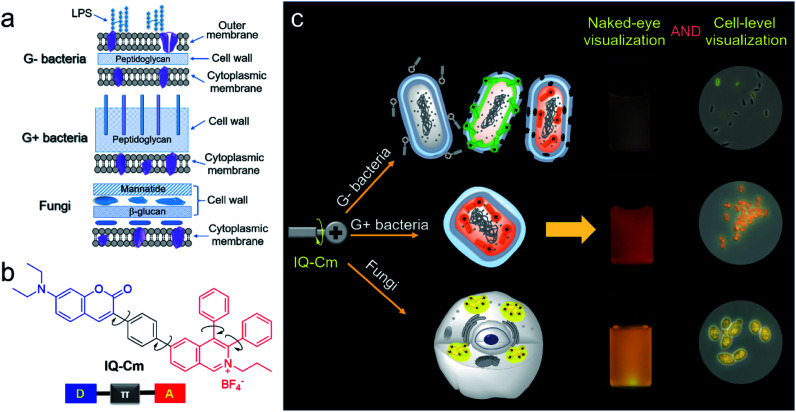
(a) Schematic of cell envelope structures of Gram-negative (G−) bacteria, Gram-positive (G+) bacteria and fungi. (b) The chemical structure of IQ–Cm. (c) Schematic illustration of IQ–Cm for visual discrimination of the three pathogens.

Fluorescence is a promising visual tool for rapid and reliable identification of pathogens^18–21^ because it exhibits more than a one thousand times improvement in sensitivity than colorimetry.^22^ Gram-negative (G−) bacteria, G+ bacteria and fungi have different surface structures and chemical components (Scheme 1a),^6,23,24^ which enable us to visually discriminate them using fluorescence probes. G+ bacteria and fungi only have a cytoplasmic membrane covered by a loose and poriferous cell wall. In contrast, G− bacteria possess an additional outer membrane, which performs the barrier function.^23,24^ Meanwhile, different from bacteria, fungi are eukaryotic organisms and contain multiple organelles in their cell protoplasm.^25^ The difference in surface structures and chemical components of the three types of pathogens allows fluorescence probes to penetrate their cell membrane and thus localizes them in different microenvironments. A lot of fluorophores with donor (D)–acceptor (A) structures are microenvironment-sensitive and show emission color change in response to microenvironmental variation based on the twisted intramolecular charge transfer (TICT) effect.^26–28^ However, with rigid and planar molecular structures, traditional fluorescence probes generally show strong fluorescence background, poor photostability and the aggregation-caused quenching (ACQ) effect, greatly compromising their advantages in sensitivity.^29,30^ Thus, it is difficult to grasp the tiny difference among the three types of pathogens and achieve visual discrimination of them based on traditional fluorophores.

Diametrically opposed to traditional fluorophores, luminogens with aggregation-induced emission characteristics (AIEgens) provide a good solution for pathogen identification. With rotor–stator structures, they generally exhibit weak emission in solution but become highly emissive when the intramolecular motions of rotors are restricted by the surroundings.^31,32^ To date, AIEgens have enjoyed great successes in bioanalyte sensing with the merits of low background, high sensitivity and good photobleaching resistance.^33–39^ The multi-rotor structures of AIEgens also endow them with high sensitivity to the surrounding microenvironment. In particular, when bearing twisted D–A structures, AIEgens can adapt different molecular configurations and show diverse fluorescence color responses to the microenvironments based on the TICT effect.^27,40^ Moreover, the visualization of these fluorescence response colors is guaranteed because the nonradiative relaxation of the TICT state can be effectively suppressed by the AIE properties.^41,42^ Thus, rationally integrating the merits of AIE and the TICT effect into one fluorophore is very promising for rapid and visual identification of the three pathogens.

In this work, we designed and prepared a new cationic AIE-active molecule with a twisted and extended donor–π–acceptor (D–π–A) structure, named IQ–Cm, for visual identification of pathogen types. Structurally, IQ–Cm consists of three parts: a diphenyl isoquinolinium (IQ) unit, a coumarin-derived (Cm) moiety and a phenyl linker (Scheme 1b). The IQ moiety has a highly twisted molecular structure and was introduced as an AIE-active group.^43^ Also, its intrinsic cationic structure allows IQ to act as a strong electron acceptor and a targeting group for the negative pathogen surface. Since many coumarin derivatives are membrane-active,^44^ coumarin was introduced to help IQ–Cm effectively interact with the pathogen membrane. A diethylamino group was attached on the coumarin to serve as a strong electron donor. Then, a rotatable aromatic phenyl was employed as the linker between IQ and Cm to generate an extended and twisted D–π–A structure,^42^ which endows IQ–Cm with prominent AIE and TICT properties. Driven by the intrinsic structural differences in the outer envelopes and cytoplasm components of the three pathogens, IQ–Cm selectively locates in different sites in them, senses the diverse surrounding microenvironment, and thus successfully transforms pathogen information into distinctive fluorescence colors at the cellular level (Scheme 1c), achieving fast discrimination of them by the naked-eye. Furthermore, we also demonstrated the potential of IQ–Cm in fast pathogen diagnosis in practice, such as fast UTI diagnosis, visual monitoring of hospital-acquired infections and naked-eye detection of molds.

## Results and discussion

### Synthesis and photophysical properties of IQ–Cm

**Scheme 2:**
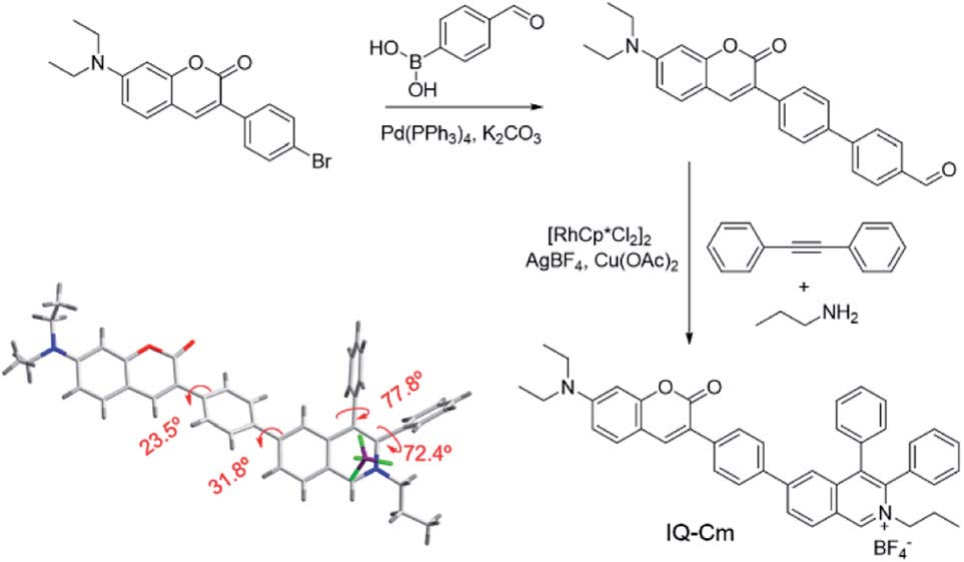
Synthetic route and single crystal structure of IQ–Cm.

The synthesis of IQ–Cm was readily achieved through two sequential steps of Suzuki coupling and a one-pot multiple component reaction with a high yield of 84% (Scheme 2). The detailed synthesis procedures are described in the ESI.† The chemical structure of IQ–Cm was completely characterized by ^1^H NMR, ^13^C NMR, and HRMS (Fig. S1–S3†) and confirmed by X-ray single crystal analysis (Scheme 2 and Table S1†).

The single crystal structure demonstrates that IQ–Cm adopts a twisted 3D conformation with large torsional angles for the aromatic groups, *i.e.* 72.4–77.8° torsion of phenyl groups from the isoquinolinium core and 23.5–31.8° torsion of coumarin and the isoquinolinium moiety from the phenyl linker (Scheme 2). These rotatable aromatic units would effectively prevent the detrimental π–π stacking, overcoming the ACQ effect. As demonstrated, the distance between the nearest two coumarin or two isoquinolinium planes is about 11 Å (Fig. S4†), much larger than that of prominent π–π interaction (3.3–3.8 Å).^45^ Multiple intermolecular hydrogen bonds C–H⋯F were found in the crystal packing (Fig. S4†), which restrict the motion of aromatic rotors. As a result, the IQ–Cm crystal gives intensive orange-red emission at 620 nm with a fluorescence quantum yield (*Φ*_F_) of 5.7%.

**Fig. 1:**
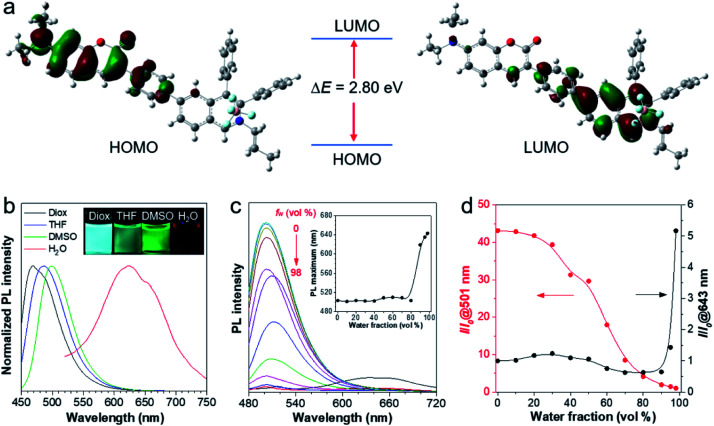
(a) The frontier molecular orbitals (HOMO and LUMO) of the ground state of IQ–Cm. (b) Normalized photoluminescence (PL) spectra of IQ–Cm (10 μM) in dioxane (Diox), THF, DMSO and water with 1% DMSO. *E*_x_: 430 nm. (c) PL spectra of IQ–Cm (20 μM) in DMSO/H_2_O mixtures with different water fractions (*f*_w_). *E*_x_: 450 nm. Inset: plots of the PL maximum wavelength *versus* water fraction in DMSO/H_2_O mixtures. (d) Plots of relative PL intensity (*I*/*I*_0_) at 501 or 643 nm *versus* water fraction in DMSO/H_2_O mixtures, where *I*_0_ at 501 nm and *I*_0_ at 643 nm refer to the PL intensity at 98% *f*_w_ and 0% *f*_w_, respectively.

To better understand the molecular characteristics, the electron cloud on the frontier molecular orbitals of IQ–Cm was calculated by density functional theory. It was found that the electron clouds of the highest occupied molecular orbital (HOMO) and lowest unoccupied molecular orbital (LUMO) of IQ–Cm are almost completely separated (Fig. 1a). Its HOMO is mainly contributed by the electron-donating *N*,*N*-diethylamino-coumarin unit, while the LUMO is mainly localized on the electron-withdrawing isoquinolinium part. This massive shift of the electron cloud means the occurrence of the obvious TICT upon excitation.^43^ This feature endows IQ–Cm with a prominent sensitivity to microenvironmental polarity variation. As shown in Fig. 1b, under UV light irradiation, IQ–Cm presents a remarkable solvatochromism effect. When changing the solvent from dioxane to water, the emission color of the IQ–Cm solution changes from blue (469 nm) to red (625 nm). In contrast, the absorption of IQ–Cm shows little dependence on the solvent polarity, only varying from 399 nm to 437 nm with an extinction coefficient of about 37 000 M^−1^ cm^−1^ in DMSO (Fig. S5†). This obvious change in the emission color of IQ–Cm in response to the environmental polarity greatly favors it for visually identifying pathogens.

In addition, IQ–Cm also exhibits AIE properties due to its highly twisted configuration. The *Φ*_F_ of IQ–Cm in the solid state (14.6%) is about 18 fold greater than that in DMSO solution (0.8%), showing the typical AIE characteristics. To further illustrate the AIE properties of IQ–Cm, it was studied in DMSO/water mixtures with different water fractions (*f*_w_). As shown in Fig. 1c and d, when increasing the water content from 0 to 80%, the emission intensity of IQ–Cm at 501 nm gradually decreases and the emission maximum slightly redshifts (Fig. 1c, inset), due to the more polar environment. On further increasing the water content from 80% to 98%, a large red-shifted emission at 643 nm appears and increases (Fig. 1c and d), showing an AIE phenomenon because of the formation of aggregates. As confirmed, with the increase of water fractions above 80%, the scattering intensity of the IQ–Cm solution abruptly increases, indicating the occurrence of aggregation (Fig. S6a†). The DLS result and TEM image show that IQ–Cm forms rod aggregates of about 1 μm (Fig. S6b and c†). This effectively restricts the motion of the aromatic rotors of IQ–Cm and activates its AIE process. This AIE effect dominates over the TICT effect and resists the emission drop caused by nonradiative relaxation of the TICT state, giving the boosted red-shifted emission of the TICT state.^41,42^ As confirmed in Fig. S6d,† with the increase in the solvent viscosity, TICT emission at 600 nm appears and gradually increases despite enhanced ICT emission at 500 nm. This further indicates that the restriction of the motion of the aromatic rotors of IQ–Cm can inhibit the non-radiative pathways of the ICT and TICT states and enhance their fluorescence. Additionally, because IQ–Cm aggregates are in the amorphous state (Fig. S6e†), their emission is vulnerable to the surrounding solvent polarity, resulting in the continued red-shift of the emission maximum after aggregation at water fractions above 80% (Fig. 1c, inset).^42^ Furthermore, due to the formed loose and amorphous structure, IQ–Cm in the aggregated state still shows weak emission (Fig. 1c), which is conducive for low background. Also, IQ–Cm has a good photostability and shows almost no signal loss at a concentration of 10 μM after continuous irradiation for 60 scans and only about 10% signal loss when the concentration decreases to 1 μM (Fig. S7†), which is comparable to that of the commercial dyes propidium iodide (PI) and MitoTracker Green. These desired properties of IQ–Cm are highly suitable for the visual identification of pathogens as discussed in the following.

### Visual identification of pathogens using IQ–Cm by the naked-eye

**Fig. 2:**
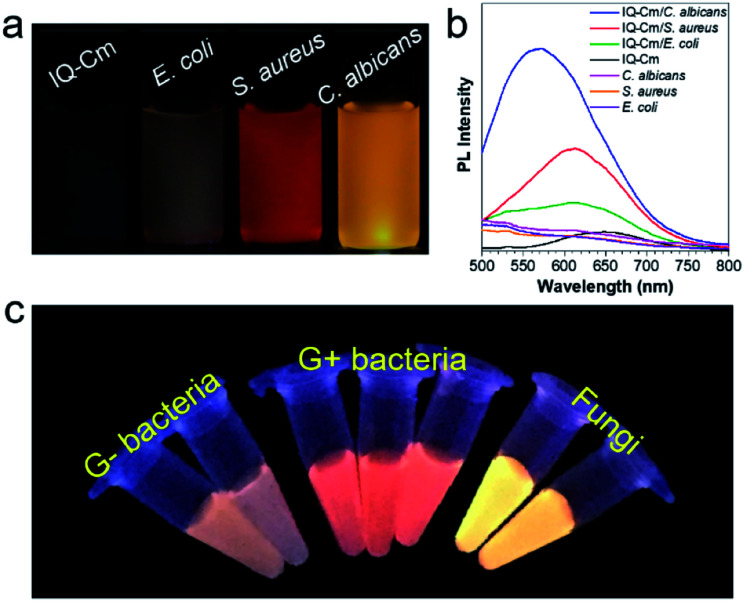
(a) Photographs of IQ–Cm and IQ–Cm with different pathogens taken under 365 nm UV irradiation ([*E. coli*] = 10^8^ CFU mL^−1^, [*S. aureus*] = 10^8^ CFU mL^−1^ and [*C. albicans*] = 10^7^ CFU mL^−1^). (b) Fluorescence spectra of 10 μM IQ–Cm in PBS solution with and without pathogens and pathogens alone in PBS solution, *E*_x_: 450 nm. (c) Photographs of IQ–Cm with different pathogens in PBS solutions obtained under 365 nm UV irradiation (from left to right in photographs: *P. aeruginosa*, *E. coli*, *S. aureus*, *E. faecalis*, *B. subtilis*, *C. albicans* and *S. cerevisiae*).

*E. coli* (G− bacteria), *S. aureus* (G+ bacteria) and *C. albicans* (fungi), the three most common pathogens in clinical environments, were chosen as representatives for demonstration. To enable naked-eye identification, 10 μM IQ–Cm in PBS solution was used as the working solution. Unlike in the case of water, where rod aggregates are formed (Fig. S6c†), IQ–Cm forms more loose network structures above its critical aggregation concentration of ∼4 μM in PBS (Fig. S8†). As a result, IQ–Cm shows a weaker fluorescence background in PBS, which is favorable for the high sensitivity and accuracy of pathogen identification. As shown in Fig. 2a, under a UV lamp, the fluorescence of IQ–Cm in PBS solution is negligible. After incubation with the three pathogens, the fluorescence emission of IQ–Cm is obviously enhanced with three distinguishable emission colors (Fig. 2a). *E. coli* shows weak pink fluorescence, *S. aureus* presents brighter orange-red fluorescence while *C. albicans* gives the strongest yellow emission. The corresponding fluorescence spectra and those of the pathogens themselves in PBS solution were also recorded (Fig. 2b). The three pathogens show weak auto fluorescence and cause a variation of emission intensity of IQ–Cm following the order of *C. albicans* > *S. aureus* > *E. coli*. A large blue-shift from 650 nm to 575 nm is induced by *C. albicans* and a smaller blue-shift to 610 nm is caused by *S. aureus*. The addition of *E. coli* causes a blue-shift of the IQ–Cm emission with two peaks centered at about 535 and 610 nm.

In order to further prove the feasibility of this naked-eye identification method, more pathogens were chosen to be treated with IQ–Cm. Similar fluorescence responses were observed for pathogens of the same kind (Fig. 2c and S9a†), *i.e.*, weak pink for G− bacteria, orange-red for G+ bacteria and strong yellow for fungi. The visual identification sensitivity of the three pathogens using IQ–Cm is about 10^8^, 10^8^, and 10^7^ CFU mL^−1^ for G− bacteria, G+ bacteria and fungi, respectively (Fig. S9b†). These results explicitly demonstrate that IQ–Cm is highly suitable for naked-eye discrimination and identification of G− bacteria, G+ bacteria and fungi by giving three distinct fluorescence colors.

### Mechanism of visual identification of pathogens

**Fig. 3:**
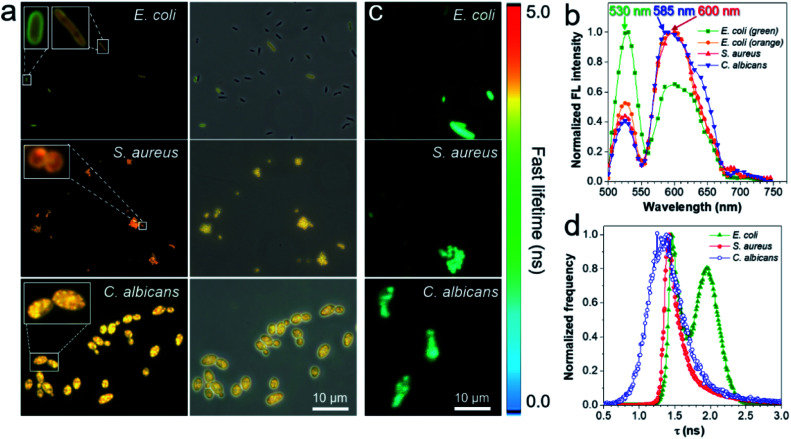
(a) Fluorescence images of *E. coli*, *S. aureus* and *C. albicans* incubated with IQ–Cm for 10 min. Excitation filter = 460–490 nm. (b) Fluorescence spectra of the three pathogens stained with IQ–Cm collected in the wavelength scanning mode of CLSM. *E*_x_: 488 nm. (c) Fluorescence lifetime imaging microscopy (FLIM) images and (d) fluorescence lifetime histogram of the three pathogens stained with IQ–Cm excited at 450 nm. [IQ–Cm] = 10 μM.

To gain insight into the fluorescence color response of IQ–Cm to the three pathogens, the fluorescence imaging technique was employed to directly visualize the interaction of IQ–Cm with the three pathogens at the cellular level. As shown in Fig. 3a, detectable weak fluorescence with low labeling efficiency is observed for *E. coli*, while *S. aureus* and *C. albicans* present bright fluorescence with high labeling efficiency, confirming the different binding affinities of IQ–Cm to the three pathogens. Similar imaging results were also observed for other pathogens of the same kind (Fig. S10†). The three pathogens labeled at the cell level show different emission colors, *i.e.,* green and orange for *E. coli*, orange for *S. aureus* and yellow for *C. albicans*. Their *in situ* fluorescence spectra were measured and they further confirmed these different emission colors (Fig. 3b and S11†). As shown, when IQ–Cm mainly stains the cell membrane of *E. coli*, the main emission peak is at 530 nm with green emission (Fig. 3a, b, S11a and d†). However, with more IQ–Cm entering the cytoplasm of *E. coli*, the main emission peak is red-shifted to 600 nm with orange emission. Different from *E. coli*, IQ–Cm mainly locates in the cytoplasm of *S. aureus* and *C. albicans* (Fig. 3a, S11b and c†) and gives an orange emission at 600 nm for *S. aureus* and a yellow emission at 585 nm for *C. albicans* (Fig. 3b and S11d†). These *in situ* spectra on the cell level from the fluorescence microscope are almost consistent with those of their bulk solutions (Fig. 2b). The above imaging results and *in situ* spectra reveal that IQ–Cm selectively interacts with the three pathogens and locates in different sites, which leads to its discernible emission colors in the three pathogens.

Furthermore, to obtain more information on IQ–Cm in the three pathogens, fluorescence lifetime imaging was performed (Fig. 3c), based on the fact that the fluorescence lifetime of a fluorophore relies more on its local environment but less on other variables such as the excitation intensity and the local fluorophore concentration.^46^ Two populations with distinct lifetimes of about 1.45 and 1.97 ns were observed for labeled *E. coli* (Fig. 3d), corresponding to IQ–Cm in the cytoplasm of *E. coli* and IQ–Cm in the cell membrane of *E. coli*, respectively (Fig. S12†). In contrast, IQ–Cm in *S. aureus* shows one lifetime of about 1.42 ns. In the case of *C. albicans*, the lifetime of IQ–Cm is widely distributed but mainly at about 1.34 ns. The distinct lifetime means that IQ–Cm experiences different microenvironments in the three pathogens. In response to the difference in the local environment, IQ–Cm adopts different twisted molecular configurations in the three pathogens, giving different emission colors based on its TICT effect discussed above. These results fully demonstrate that IQ–Cm shows different interactions with the three pathogens and selectively lies in different microenvironments.

**Fig. 4:**
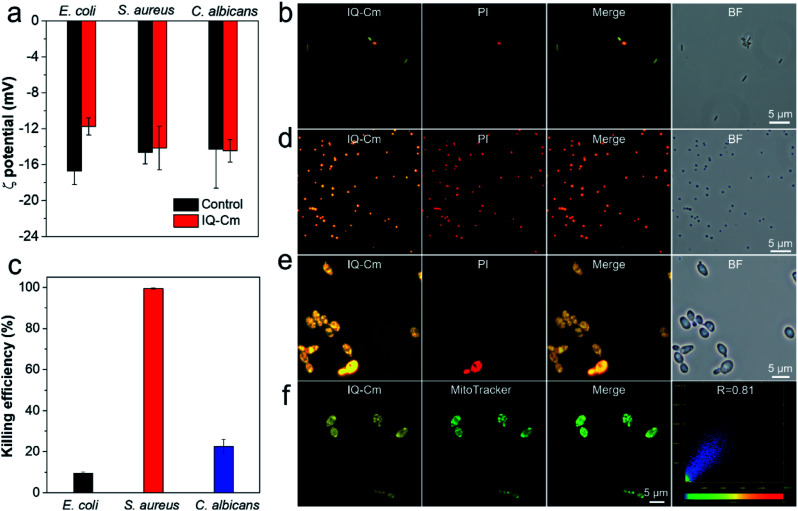
(a) Zeta potential results of the three pathogens before and after incubation with 10 μM IQ–Cm in PBS solution. (b, d and e) Fluorescence microscope images of *E. coli*, *S. aureus* and *C. albicans* stained with 10 μM IQ–Cm and 5 μg mL^−1^ PI in PBS solution for 10 min, respectively. Imaging conditions: excitation filter = 460–490 nm for IQ–Cm and 510–550 nm for PI. (c) Antimicrobial activity of 10 μM IQ–Cm toward the three pathogens. (f) Confocal fluorescence images of *C. albicans* stained with 1 μM IQ–Cm and 100 nM MitoTracker Green in PBS solution for 10 min. Conditions: *λ*_ex_ = 405 nm and *λ*_em_ = 600–620 nm for IQ–Cm. *λ*_ex_ = 488 nm and *λ*_em_ = 500–520 nm for MitoTracker Green.

Next, we further explored the mechanism behind the diversity of interactions and color responses of IQ–Cm to the three pathogens. To understand the lower labeling efficiency of IQ–Cm to G− bacteria than to the other two pathogens, we first investigated their cell envelope structure. As shown in Scheme 1a, compared with G+ bacteria and fungi, G− bacteria possess an additional outer membrane, which exhibits the barrier function.^23,24^ Therefore, IQ–Cm is effectively prevented from accessing the cytoplasmic membrane of live G− bacteria. In contrast, lacking the protection of an outer membrane, IQ–Cm can readily penetrate the cell membrane and enter the inside of G+ bacteria and fungi driven by its cationic (IQ) and membrane-active (Cm) groups (Fig. 3a, S11b and c†). As demonstrated by the zeta potential results (Fig. 4a), after adding IQ–Cm, the surface potentials of *S. aureus* and *C. albicans* do not obviously change while that of *E. coli* becomes more positive. This indicates that IQ–Cm primarily enters the inside of *S. aureus* and *C. albicans* but attaches to the surface of *E. coli via* electrostatic interactions. The cationic IQ–Cm compromises the negative potential of the surface of *E. coli*.^6^ After entering *S. aureus* and *C. albicans*, the intramolecular motions of the aromatic rotors of IQ–Cm are restricted effectively by the internal environment, which turns on its emission based on the working mechanism of AIEgens–restriction of intramolecular motion (RIM).^31^ As a result, IQ–Cm shows high labeling efficiency for *S. aureus* and *C. albicans*, which accounts for the strong emission of their bulk suspension. But the aromatic rotors of IQ–Cm on the surface of *E. coli* undergo motion with little restriction, making it almost nonemissive. Thus, only a few *E. coli* with a compromised outer membrane or destroyed cell membrane (dead *E. coli*) allow IQ–Cm to insert or penetrate their cell membrane, which contributes to the low labeling efficiency by IQ–Cm and the weak emission of the IQ–Cm/*E. coli* bulk suspension.

To verify our claim, co-staining experiments were performed for *E. coli* using IQ–Cm and propidium iodide (PI, a specific probe for dead microbes with a destroyed cell membrane along with red emission^47^). As shown in Fig. 4b, the red emission of PI is observed for orange *E. coli* but not for green ones. Based on the fact that PI selectively enters the cell protoplasm of dead microbes with destroyed cell membranes,^47^ the co-staining results reveal that dead *E. coli* are labeled with IQ–Cm, which gives orange emission. Meanwhile, the antibacterial results show that IQ–Cm has about 10% killing activity against *E. coli*, indicating the existence of a few dead *E. coli* (Fig. 4c). To further prove this, *E. coli* were killed by medical alcohol to destroy their cell membranes. Evidently, for *E. coli* treated with medical alcohol, IQ–Cm shows high staining efficiency and enters their cell protoplasm, giving orange emission (Fig. S13†). These results fully confirm that IQ–Cm destroys the cell membrane of a few *E. coli* and thus is allowed to enter their cell protoplasm. Unlike PI that specifically binds with the nucleic acid in the cell protoplasm,^47^ IQ–Cm possibly just locates in the cell protoplasm, as the addition of DNA and RNA can obviously enhance the emission of PI rather than IQ–Cm (Fig. S14†). It has been reported that the cell cytoplasm of bacteria contains a large amount water (∼80%)^48^ and presents glass-like properties.^49^ Hence, in response to such a largely polar and rigid microenvironment, IQ–Cm gives a red-shifted emission of orange color due to the TICT and AIE effects.

On the other hand, red emission was not observed for the *E. coli* with green fluorescence (Fig. 4b), which suggests that the outer membrane of these *E. coli* was possibly destroyed but the cytoplasmic membrane was intact. To verify this, we destroyed the outer membrane of *E. coli* and kept their cytoplasmic membrane intact by adding ethylenediaminetetraacetate disodium (EDTA) to remove Ca^2+^ or/and Mg^2+^ which control the integrity of the outer membrane.^50^ As shown in Fig. S15,† IQ–Cm exhibits high staining efficiency for treated *E. coli*, where *E. coli* with the hollow green emission is observed. This indicates that IQ–Cm primarily locates at the cell membrane of the *E. coli* with the compromised outer membrane but intact cytoplasmic membrane. As the cell membrane mainly consists of various lipids with a low surrounding polarity,^51^ a blue-shifted green emission of IQ–Cm is exhibited due to the TICT effect. Taken together, with the barrier of the outer membrane, IQ–Cm primarily targets the negative surface of *E. coli*, and thus a major of *E. coli* are almost nonemissive. Only a small portion of *E. coli* with a compromised outer membrane or dead *E. coli* are lit up by IQ–Cm, where IQ–Cm molecules mainly insert into the cell membrane of *E. coli* with compromised outer membranes and enter the cell protoplasm of dead *E. coli*. In response to the difference in these two microenvironments of *E. coli*, IQ–Cm gives green and orange emission due to the TICT effect. These above factors contribute to two fluorescence colors with low labeling efficiency under fluorescence microscopy and weak pink fluorescence color observed by the naked-eye.

Besides, the co-staining experiments with IQ–Cm and PI were also performed for the G+ *S. aureus* and fungal *C. albicans*. Clearly, both the orange signal from IQ–Cm and red signal from PI were observed for most of the G+ *S. aureus* (Fig. 4d). This means that IQ–Cm shows strong interaction with *S. aureus* and completely kills them, as proved by the nearly 100% killing efficiency of IQ–Cm to *S. aureus* (Fig. 4c). Similarly to dead *E. coli*, in the largely polar and glass-like microenvironment of the cell cytoplasm, IQ–Cm gives a red-shifted emission of orange color in *S. aureus* based on the TICT and AIE effects.

Meanwhile, fungal *C. albicans* presents a different scenario. As shown in Fig. 4e, low staining efficiency from PI was observed, suggesting that IQ–Cm shows low killing activity against *C. albicans* (Fig. 4c). Both dead and live *C. albicans* give a similar yellow emission as there is no barrier of the additional outer membrane and IQ–Cm can easily enter the cytoplasm of fungi regardless of the viability. Moreover, fungi are eukaryotic organisms and contain multiple organelles such as mitochondria, the endoplasmic reticulum and the Golgi apparatus in their cell protoplasm.^25^ Inspired by the fact that cationic AIEgens possess good specificity to the mitochondria of eukaryotic cells,^43,52^ we hypothesize that the cationic IQ–Cm could also target and accumulate in the mitochondria of fungi. To confirm this, colocalization of IQ–Cm and MitoTracker Green (a commercial probe for mitochondrial imaging of yeast) was performed. As shown in Fig. 4f and S16,† the yellow emission from IQ–Cm and the green emission from MitoTracker Green are almost completely overlapping with a high Pearson's correlation coefficient of 0.81, confirming that IQ–Cm mainly locates in the mitochondria of fungi. Compared with the cell membrane, the mitochondrial membrane contains a greater membrane protein content,^53^ which gives rise to a more polar environment than that of the cell lipid membrane but still less than that of the cell protoplasm with a large amount water. Thus, an intermediate yellow emission was observed.

Above all, IQ–Cm shows diverse interaction with the three pathogens and selectively locates in different sites. Accordingly, in the three pathogens, IQ–Cm experiences different surrounding microenvironments and finally gives three discernible fluorescence colors, achieving visual discrimination (Scheme 1c).

### Fast diagnosis of urinary tract infections (UTIs)

**Fig. 5:**
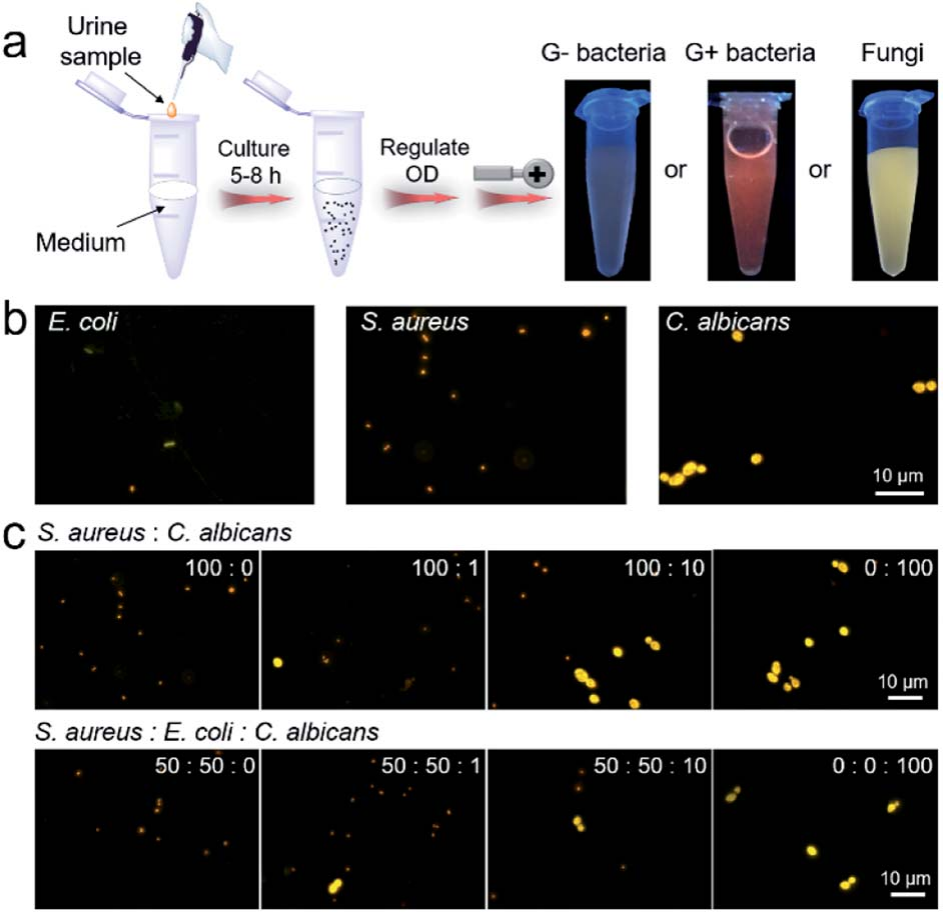
(a) The procedures and results by naked-eye identification of built UTI samples based on IQ–Cm (OD is the optical density of the pathogen suspension at 600 nm). (b) Fluorescence images of built urine samples stained with IQ–Cm for 10 min. (c) Fluorescence images of urine samples with different bacterium/fungus combinations and ratios stained with IQ–Cm for 10 min (the corresponding bright field images with the three pathogens are shown in Fig. S17†).

The high efficiency of IQ–Cm for visual pathogen identification inspires us to employ it for clinical diagnosis. UTIs are one of the most common pathogen infections of humans^5^ and is thus chosen as a representative example. In clinics, urine culture is recommended as the gold standard for UTI diagnosis, but it generally takes several days.^54^ Based on the above results, IQ–Cm shows high potential for fast diagnosis of UTIs. To validate this, UTI models were built by adding *E. coli*, *S. aureus* and *C. albicans* into normal urine to mimic clinical G− bacterial, G+ bacterial and fungal infections. Firstly, these UTI model samples were visually identified by the naked eye. As shown in Fig. 5a, 10 μL infected urine samples were transferred to 10 mL culture medium and then grown for about 5–8 h. This culture step can effectively reduce the interference of the complex components in a patient's urine. The collected pathogens were incubated with IQ–Cm for 10 min and then directly observed under a UV lamp. The sample with weak pink color was identified as G− bacterial infection, orange-red color as G+ bacterial infection and bright yellow color as fungal infection. These identified results were consistent with the original added pathogen type, conceptually demonstrating the feasibility of IQ–Cm for UTI diagnosis. Although microbial culturing is required, this naked-eye visual identification method is simple and only takes a few hours, which is much faster than the several days of traditional urine culture.

These fabricated UTI model samples were also visually identified using a fluorescence microscope. After simply centrifuging the urine samples and resuspending the collected pathogens in PBS, IQ–Cm was added for 10 min, and then the samples were observed under a fluorescence microscope. Based on the distinctive fluorescence response of the three pathogens to IQ–Cm at the cellular level, correct identification results were obtained within 30 min (Fig. 5b).

Another noteworthy issue is that hospitalized patients often receive hospital-acquired infections due to their compromised immunity caused by the use of broad-spectrum antibiotics or immunosuppressive agents.^55–57^ As a worse case, the initial bacterial infection of patients may evolve into a fungal infection when post-operative antibiotics are inappropriately used.^58^ Based on the discernible fluorescence response of IQ–Cm to bacteria and fungi, this hospital-acquired infection process can be easily monitored using IQ–Cm, which is very conducive for clinical decisions. To simulate the occurrence and evolution of opportunistic fungal UTIs from an initial bacterial infection in a hospital, UTI models were built by adding different number ratios of bacteria (*S. aureus*/*E. coli*) and fungi (*C. albicans*) into normal urine. As shown in Fig. 5c, under a fluorescence microscope, the emergence of a very small amount of fungal species in bacterial communities could be noticeably observed, due to their intensely yellow emission. As the fungal numbers gradually increase and exceed that of bacteria, *i.e.*, the number ratios of bacteria and fungi from 100 : 0, 100 : 1, and 100 : 10 to 0 : 100, the species with bright yellow emission become greater in number and finally become dominant. This means that the cause of infection has been completely changed, and thus the antimicrobial formula should be adjusted accordingly. For a more complicated situation, where the initial infection was caused by two kinds of bacteria, the emergence of fungal species can also be easily monitored (Fig. 5c). These results fully demonstrate the high potential of IQ–Cm for fast clinical diagnosis and timely monitoring of hospital-acquired infection.

### Visual detection of mold

**Fig. 6:**
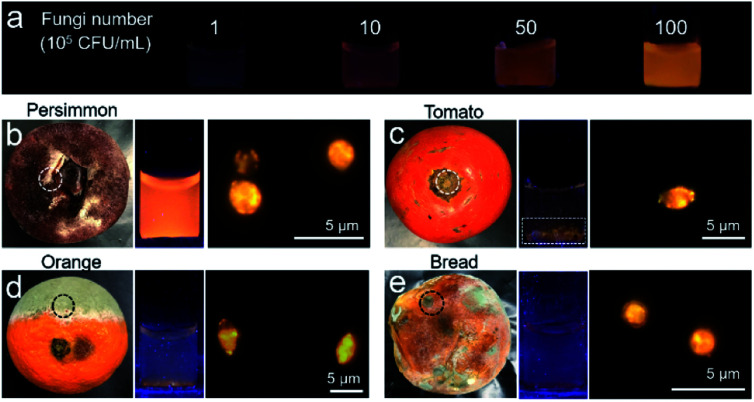
(a) The photographs of IQ–Cm obtained under 365 nm UV irradiation before and after adding *C. albicans* with different concentrations. (b–e) The tested samples and the photographs of IQ–Cm under 365 nm UV irradiation after adding the molds, and fluorescence images of molds stained with IQ–Cm. Among them: (b) mold collected from persimmon in the white-dotted circle. (c) Mold from tomato after removing the rotten stem in the white-dotted circle. (d) Mold from orange in the black-dotted circle. (e) Mold from bread in the black-dotted circle. [IQ–Cm] = 10 μM.

The feasibility of IQ–Cm in detecting molds was also explored. Molds are a type of fungus and can be seen everywhere in our life.^59^ Unavoidably, tiny spores of molds floating in the air can fall onto food or food processing equipment and grow into molds, which greatly threaten human health.^60^ Thus, the rapid detection of molds is very important. The bright yellow emission of fungi labeled with IQ–Cm greatly facilitates the detection of molds. Moreover, the mold number can also be roughly quantified by the naked-eye under a UV lamp. To demonstrate this, an emission color-fungal amount relationship was first established (Fig. 6a). It was found that the limit of naked-eye detection of IQ–Cm for fungi is about 10^6^ CFU mL^−1^. This means that when the fluorescence emission can be observed by the naked-eye, the fungal number is beyond 10^6^ CFU mL^−1^. Above the detection limit, the emission color changes from light coral and orange to yellow, corresponding to the change in fungal amount from 10^6^ and 5 × 10^6^ to 10^7^ CFU mL^−1^, respectively. This emission color-fungal amount relationship was also verified by PL spectroscopy (Fig. S18†).

Based on the established emission color-fungal amount relationship, the mold amount grown on food can be easily and visually determined by the naked-eye. To demonstrate this, four classes of representative foods involving preserved fruit (persimmon), vegetable (tomato), fruit (orange) and wheaten food (bread) were taken as the test samples. In appearance, the persimmon did not look moldy, the stem of the tomato was rotten, and the orange and bread were obviously moldy. By naked-eye detection and fluorescence microscope imaging, all four samples were detected to have mold growth (Fig. 6b–e). But the emission colors for the four samples were obviously different from each other. According to the emission color-fungal amount relationship, it can be concluded that the mold number grown on the chosen four samples follows the order of persimmon > tomato ≈ orange > bread (specifically, ∼10^7^ CFU mL^−1^ for persimmon, 10^6^ to 10^7^ CFU mL^−1^ for tomato and orange, and ∼10^6^ CFU mL^−1^ for bread). Interestingly, this result does not agree with the observed apparent phenomena. For the obviously moldy orange and bread, the detected mold amount using IQ–Cm is less. This is reasonable because the observed massive “molds” are mainly mycotoxins produced by molds.^59^ But there is still a small number of molds hiding among them, as detected using IQ–Cm (Fig. 6d and e). Conversely, the persimmon and tomato with the rotten stem removed seem to have no mold, but a large number of molds are detected using IQ–Cm (Fig. 6b and c). These results suggest that the seemingly benign food possibly hides a large number of molds, just like the situation of the persimmon. In this case, IQ–Cm can rapidly “see” these molds, which is essential for monitoring food quality.

## Conclusions

In conclusion, we have rationally designed a simple AIEgen with a twisted D–π–A structure, which serves as a microenvironment-sensitive probe for rapid visual discrimination of G− bacteria, G+ bacteria and fungi by giving discernible emission colors. IQ–Cm successfully identifies the subtle differences between the three pathogens by primarily locating in different sites, *i.e.* cell envelop and cell cytoplasm of G− bacteria, cell cytoplasm of G+ bacteria and mitochondria of fungi. In these three cases, IQ–Cm experiences diverse surrounding environments and thus effectively transforms the pathogen information into distinctive fluorescence colors due to its AIE properties and TICT effect. At the naked-eye level, G− bacteria are weak pink, G+ bacteria are orange-red and fungi are bright yellow, aiding direct and fast discrimination of them. With a fluorescence microscope, the visual discrimination of the three pathogens is realized at the cell level on the basis of their specific fluorescence response. More importantly, IQ–Cm shows high potential for fast diagnosis of UTIs, which can reduce the diagnosis time to a few hours by direct naked-eye detection or less than 30 minutes using a fluorescence microscope. Also, based on the distinct fluorescence color response of IQ–Cm to bacteria and fungi, the frequent hospital-acquired infection evolution from an initial bacterial infection to a fungal infection can be timely and visually monitored using IQ–Cm. This is very essential for guiding clinical decisions. Furthermore, thanks to the bright yellow emission of labeled fungi, IQ–Cm can be used for fast detection of molds in the food field. Moreover, the mold number can also be roughly quantified according to the established emission color-fungal amount relationship. Therefore, our studies provide a fast and simple platform for pathogen identification at the point-of-care level, which exhibits very high potential in offering timely and reliable pathogen information for making clinical treatment decisions, monitoring the trends of infectious diseases and supervising food safety.

## Experimental section

### Materials and methods

All the chemicals and organic solvents were purchased from J&K, TCI and Sigma-Aldrich Company and used as received. Two Gram-negative (G−) bacteria, *E. coli* (ATCC25922) and *P. aeruginosa* (JCm5962), three Gram-positive (G+) bacteria, *S. aureus* (ATCC6538), *E. faecalis* (JCm5803) and *B. subtilis* (DSM2109), and two fungi, *C. albicans* (ATCC10231) and *S. cerevisiae* (P11), were obtained from the China General Microbiological Culture Collection Center and Beijing Bio-Med Technology Development Co., Ltd. Phosphate buffered saline (1× PBS, pH 7.4) was used throughout the identification work of the pathogens. NMR spectra were measured using a Bruker ARX 400 NMR spectrometer. High-resolution mass spectrometry (HRMS) measurements were performed in MALDI-TOF mode on a Finnegan MAT TSQ 7000 Mass Spectrometer. UV-vis absorption and photoluminescence (PL) spectra were recorded on a Milton Ray Spectronic 3000 array spectrophotometer and a PerkinElmer LS 55 spectrometer, respectively. The absolute fluorescence quantum yield was measured with a Hamamatsu quantum yield spectrometer C11347. The size distribution and zeta potential results were recorded on a ZetaPALS Brochure. Fluorescence images and laser confocal scanning microscope images were collected on a fluorescence microscope (Upright Biological Microscope Ni-U) and confocal laser scanning microscopy (Zeiss LSM800 and Leica SP8), respectively. Fluorescence lifetime imaging was performed using an OLYMPUS IX73 microscope system. The morphology and transmission electron diffraction (TED) pattern of IQ–Cm aggregates were observed and collected by transmission electron microscopy (TEM, JEM 2010). Theoretical calculations were carried out with Gaussian 09 software at the B3LYP/6-31G** level.

### Naked-eye identification of pathogens

IQ–Cm was added into a pathogen PBS suspension with a final concentration of 10 μM (G− bacteria (OD_600_ = 1.0), G+ bacteria (OD_600_ = 1.0) or fungi (OD_600_ = 2.0)). The mixtures were incubated for about 10 min at room temperature, and then observed under 365 nm UV irradiation.

### Pathogen staining and imaging

After incubating pathogens with IQ–Cm under the same conditions as described in the experiments of naked-eye identification of pathogens, the mixtures were concentrated about 10 times by centrifugation (7100 rpm for 2 min). 2 μL of concentrated pathogen suspension was transferred to a clean glass slide, slightly covered by a coverslip, left for about 2 min for immobilization and then imaged. Imaging conditions of the fluorescence microscope: 100× objective lens, excitation filter = 460–490 nm, dichroic mirror = 505 nm, emission filter = 515 nm long pass. For CLSM imaging using a Leica SP8: 100× objective lens, excitation filter: 488 nm, emission filter: 500–750 nm. Fluorescence lifetime imaging under an OLYMPUS IX73 microscope system: *λ*_ex_ = 450 nm with fs laser pulses and *λ*_em_ = 500–700 nm detected using a hybrid photomultiplier detector (PMA Hybrid 40, PicoQuant).

Co-staining experiment: by following the above operation steps, three pathogens were incubated with 10 μM IQ–Cm and 5 μg mL^−1^ propidium iodide (PI) in PBS solution for 10 min and then imaged. For colocalization with the commercial mitochondrial dye MitoTracker Green, *C. albicans* was incubated with 1 μM IQ–Cm and 100 nM MitoTracker Green for 10 min. Imaging conditions: in the case of co-staining with PI, the images were collected using a fluorescence microscope with excitation filter = 460–490 nm, dichroic mirror = 505 nm, emission filter = 515 nm long pass for IQ–Cm and with excitation filter = 510–550 nm, dichroic mirror = 570 nm, emission filter = 590 nm long pass for PI. For colocalization with MitoTracker Green in *C. albicans*, the images were obtained using a Zeiss 800 confocal microscope with excitation wavelength *λ*_ex_ = 405 nm and emission wavelength *λ*_em_ = 600–620 nm for IQ–Cm and *λ*_ex_ = 488 nm and *λ*_em_ = 500–520 nm for MitoTracker Green.

## Conflicts of interest

There are no conflicts to declare.

## Supplementary Material

SC-011-D0SC00256A-s001

SC-011-D0SC00256A-s002
