# Supplementary material for: One stone, three birds: one AIEgen with three colors for fast differentiation of three pathogens
Source: Chem Sci. 2020 Apr 14;11(18):4730–40. doi: 10.1039/d0sc00256a (PMC8159167; doi:10.1039/d0sc00256a)
Supplement: SC-011-D0SC00256A-s001 [file SC-011-D0SC00256A-s001.pdf]

Electronic Supplementary Material (ESI) for Chemical Science.
This journal is © The Royal Society of Chemistry 2020

Supporting Information

# One Stone, Three Birds: One AIEgen with Three Colors for Fast Differentiating Three Pathogens

*Chengcheng Zhou,*†[ad] *Meijuan Jiang,*†[bca] *Jian Du,*[e] *Haotian Bai,*[ad] *Guogang Shan,*[ad] *Ryan T. K. Kwok,*[ad] *Joe H. C. Chau,*[a] *Jun Zhang,*[a] *Jacky W. Y. Lam,*[ad] *Peng Huang*[b] *and Ben Zhong Tang**[adf]

[a]Department of Chemistry, Hong Kong Branch of Chinese National Engineering Research Center for Tissue Restoration and Reconstruction, Department of Chemical and Biological Engineering, The Hong Kong University of Science and Technology, Clear Water Bay, Kowloon, Hong Kong, China

[b]Guangdong Key Laboratory for Biomedical Measurements and Ultrasound Imaging, Laboratory of Evolutionary Theranostics, School of Biomedical Engineering, Health Science Center, Shenzhen University, Shenzhen 518060, China

[c]Key Laboratory of Optoelectronic Devices and Systems of Ministry of Education and Guangdong Province, College of Optoelectronic Engineering, Shenzhen University, Shenzhen 518060, China

[d]HKUST Shenzhen Research Institute, No. 9 Yuexing 1st RD, South Area, Hi-tech Park Nanshan, Shenzhen 518057, China

[e]Urinary Surgery, The Affiliated First Hospital of Soochow University, Pinghai Road, Suzhou, 215006, China

[f]State Key Laboratory of Luminescent Materials and Devices, Center for Aggregation-Induced Emission (Guangzhou International Campus), South China University of Technology, Guangzhou 510640, China

†The authors contributed equally.

*Correspondence email: tangbenz@ust.hk

**Synthesis and characterization of IQ-Cm.**

The reactant 3-(4'-Bromophenyl)-7-diethylaminocoumarin was commercially available. In this work, it was synthesized according to the reported procedures.[1] Yield 55.9%. $^1$H NMR (400 MHz, $CDCl_3$), $\delta_H$ (ppm): 7.77 (s, 1H), 7.61-7.56 (m, 4H), 7.51 (d, 1H, $J$ = 8.4 Hz), 7.26 (s, 1H), 7.01 (s, 1H), 3.48 (q, 4H, $J$ = 7.2 Hz), 1.28 (t, 6H, $J$ = 7.2 Hz).

Synthesis of first step product 4'-(7-(diethylamino)-2-oxo-2H-chromen-3-yl)-[1,1'-biphenyl]-4-carbaldehyde (shown by **1** in the description below). **1** was synthesized *via* Suzuki coupling according to the reference.[2] The mixture of 3(4'-Bromophenyl)-7-diethylaminocoumarin (1.12g, 3.0 mmol), (4-formylphenyl)boronic acid (0.48 g, 3.0 mmol), $K_2CO_3$ (621 mg, 4.5 mmol) and $Pd(PPh_3)_4$ (120 mg) in 75 mL THF/water (4:1 v/v) was refluxed overnight under nitrogen. After cooling the mixture to room temperature, water was added and then extracted with dichloromethane. After removing the solvent, the crude product was further purified by silica gel column. After drying, yellow solid was obtained with 92.2% yield. Characterization: $^1$H NMR (400 MHz, $CDCl_3$), $\delta_H$ (ppm): 10.07 (s, 1 H), 7.97 (d, 2H, $J$ = 8.0 Hz), 7.84 (d, 2H, $J$ = 8.0 Hz), 7.80-7.78 (m, 3H), 7.69 (d, 2H, $J$ = 8.0 Hz), 7.35 (d, 1H, $J$ = 8.8 Hz), 6.62 (d, 1H, $J$ = 8.8 Hz), 6.55 (s, 1H), 3.45 (q, 4H, $J$ = 7.2 Hz), 1.24 (t, 6H, $J$ = 7.2 Hz); MALDI-MS calculated for $C_{26}H_{23}NO_3$ $[M]^+$: 397.1678, found: 397.1670.

Synthesis of IQ-Cm: IQ-Cm was synthesized referring to the previous report.[3] Briefly, the mixture of **1** (143.1 mg, 0.36 mmol), propylamine (0.45 mmol) and diphenylacetylene (0.30 mmol), $AgBF_4$ (58.5 mg, 0.30 mmol), $[RhCp^*Cl_2]_2$ (5.6 mg, 2.0 mol %) and $Cu(OAc)_2$ (54.5 mg, 0.30 mmol) in 2.5 mL *t*-amylalcohol was heated and stirred in protective nitrogen at 110 $^oC$ for 3 h. After removing the solvent, the residents were purified with alumina column chromatography using $CH_2Cl_2$/MeOH (100:1 v/v) as eluent. After drying, orange solids were obtained with 84% yield (155 mg). Characterization: $^1$H NMR (400 MHz, $d_6$-DMSO), $\delta_H$ (ppm): 10.28 (s, 1 H), 8.73 (d, 1H, $J$ = 8.4 Hz), 8.52 (d, 1H, $J$ = 9.6 Hz), 8.18 (s, 1H), 7.90 (d, 2H, $J$ = 8.8 Hz), 7.73 (d, 2H, $J$ = 8.4 Hz), 7.69 (s, 1H), 7.53-7.50 (m, 3H), 7.43-7.31(m, 8H), 6.76 (dd, 1H, $J$ = 8.8, 2.4 Hz), 6.57 (s, 1H), 4.40 (t, 2H, $J$ = 7.2 Hz), 3.46 (q, 4H, $J$ = 6.8 Hz), 1.85

(m, 2H), 1.14 (t, 6H, $J$ = 7.2 Hz), 0.80 (t, 3H, $J$ = 7.2 Hz); $^{13}$C NMR (100 MHz, $d_6$-DMSO), $\delta$c (ppm): 160.28, 155.97, 150.78, 149.57, 147.76, 144.37, 141.69, 138.33, 137.66, 137.21, 136.48, 133.43, 131.49, 131.01, 130.43, 130.15, 130.03, 129.90, 128.90, 128.55, 128.36, 128.31, 127.32, 125.87, 122.23, 117.36, 109.34, 108.36, 96.08, 60.25, 44.14, 23.72, 12.33, 10.50; MALDI-MS calculated for $C_{43}H_{39}N_2O_2$ $[M]^+$: 615.3006, found: 615.3018.

## Experimental methods

**Preparation of pathogen suspension.** A single pathogen colony on solid agar plate was added to 10 mL corresponding liquid culture medium (Culture medium: Luria Broth (LB) for *E. coli, P. aeruginosa*, *S. aureus* and *B. subtilis*, Tryptic Soy Broth (TSB) for *E. faecalis* and Yeast-extract Peptone Dextrose (YPD) for *C. albicans* and *S. cerevisiae*), and then grown for about 6~8 h for bacteria at 37 °C and for about 10 h for fungi at 30 °C under the shaking of 220 rpm. Pathogens were harvested by centrifuging (7100 rpm, 2 min) and washed once with PBS. The remaining pathogens were resuspended with PBS and adjusted to an optical density of 1.0 for bacteria ($OD_{600}$ = 1.0) and 2.0 for fungi ($OD_{600}$ = 2.0) at 600 nm. There are about $10^8$ CFU/mL bacteria in the solution with $OD_{600}$ of 1.0 and about $10^7$ CFU/mL fungi in the solution of $OD_{600}$ of 2.0 calibrated by spread plate method.

**Preparation and staining of cell membrane-destroyed *E. coli.*** (1) To destroy the cell membrane of *E. coli* (including the outer membrane and cytoplasm membrane), 1.0 mL *E. coli* suspension ($OD_{600}$ = 1.0) was centrifuged (7100 rpm, 2 min) and the harvested *E. coli* were treated by 200 µL of 75% alcohol for about 2 min at room temperature, then washed two times with PBS. The prepared *E. coli* with destroyed cell membrane was stained by 10 µM IQ-Cm and 5 µg/mL PI and then imaged with a fluorescence microscope. (2) For preparing *E. coli* with disrupted outer membrane but intact inner membrane, the operation was performed according to the reference[4]. Briefly, 1.5 mL *E. coli* suspension ($OD_{600}$ = 1.0) was centrifuged (7100 rpm, 2 min) and the harvested *E. coli* was washed twice using Tris–HCl (0.01 M, pH 8.0) and resuspended with 1 mL Tris–HCl buffer (0.01 M, pH 8.0, containing 0.5 M sucrose). 0.1 M potassium ethylenediaminetetraacetate (pH 8.0) was slowly added into the *E. coli* suspension with a final

concentration of 0.01 M during ~20 min. And the mixture was shaken at 220 rpm for 20 min at 37 °C to remove the outer membrane. The obtained *E. coli* with compromised outer membrane was stained with 10 μM of IQ-Cm and observed with a fluorescence microscope. The imaging conditions were same as that in the experiment of pathogen staining and imaging.

**Photostability of IQ-Cm.** The photostability of IQ-Cm was tested in time series mode (scan mode: frame, 60 frame, frame time: 5.06s) by a Zeiss 800 confocal microscope. When comparing the photostability of IQ-Cm with PI, dead *E. coli* was prepared by 75% alcohol and stained with 10 μM of IQ-Cm and PI, respectively, according to the procedure described in Preparation and staining of cell membrane-destroyed *E. coli*. The imaging slide of bacteria was prepared according to the procedures described in the Experimental section of the main text. Then the photostability of IQ-Cm and PI was tested by continuous laser light scanning under the conditions of excitation wavelength: 488 nm (0.5% laser power, pin hole 50 μm and master gain 750 V) and Em: 500-700 nm for IQ-Cm and Em: 600-700 nm for PI. For comparing the photostability of IQ-Cm with Mito-tracker Green, *C. albicans* was stained with 1 μM of dye. The photostability of IQ-Cm and Mito-tracker Green was tested by continuous laser light scanning under the conditions of excitation wavelength: 488 nm (0.8% laser power, pin hole 50 μm and master gain 750 V) and Em: 500-700 nm for IQ-Cm and Mito-tracker Green.

**Zeta potential measurements.** Three pathogens (*E. coli*, *S. aureus* and *C. albicans*) were incubated with IQ-Cm for 10 min at room temperature, respectively. The unbound IQ-Cm was removed by centrifuging (7100 rpm, 2 min). The remaining pathogens were resuspended with PBS and placed on the ice waiting for measurements. A control group of the pathogens without IQ-Cm was also performed under the same conditions.

**Dynamic light scattering (DLS) measurements.** The size distribution of IQ-Cm in DMSO/$H_2O$ mixture with 98% water and PBS solution as well as scattering intensity of IQ-Cm in DMSO/$H_2O$ mixture with different water fraction were measured on on ZetaPALS Brochure with a 35 mW red diode laser (nominal

640 nm) at a scattering angle of 173° at 25 °C.

**Cryogenic Transmission Electron Microscopy (Cryo-TEM).** 5 μL of IQ-Cm PBS solution was dripped onto a carbon coated holey TEM grids, was blotted by filter paper for three seconds, and then plugged into the liquid ethane cooled by liquid nitrogen to obtain thin layers of vitrified ice. Frozen hydrated specimens were imaged by transmission electron microscopy (TEM, JEM 2010) operated at 120 kV in low-dose mode.

**Assessment of antimicrobial activity of IQ-Cm.** The antimicrobial activity of IQ-Cm against three pathogens (*E. coli*, *S. aureus* and *C. albicans*) was evaluated by traditional surface plating method according to the reported procedures.[5]

**Fabrication and diagnosis of UTIs and hospital-acquired infection models.** UTIs models were built according to the clinical criteria of UTIs with bacteria concentration of above $10^5$ CFU/mL and fungi concentration of above $10^4$ CFU/mL.[6] About 20 mL of middle part of morning urine from healthy people was taken into sterilized 50 ml centrifuge tube. 200 μL of *E. coli* ($OD_{600}$ = 1.0), 200 μL of *S. aureus* ($OD_{600}$ = 1.0) or 200 μL of *C. albicans* ($OD_{600}$ = 2.0) solutions was first centrifuged to harvest (7100 rpm, 2 min), and then 1 mL of health urine was added to obtain the bacterial UTI models (G- bacterial infection and G+ bacterial infection) and fungal infection models. To simulate the occurrence and evolution process of hospital-acquired infection from bacterial infection to fungal infection during the patients' hospital stay, the urine samples with different number ratio of bacteria and fungi were created. Keep the total pathogen volume at 200 μL and regulate the mixed volume ratio of bacteria and fungi. In the case of urine samples with two pathogens, the chosen volume ratios of *S. aureus* and *C. albicans* ($V : V$) were gradually changed from 200 : 0, 182 : 18, 100 : 100 to 0 : 200, whose number ratio of bacteria and fungi ($N : N$) were correspondingly varied from 100 : 0, 100 : 10, 100 : 100 to 0 : 100. As for urine samples with three pathogens, where two kinds of bacteria (*E. coli* and *S. aureus*) were mixed in the equal volumes, the chosen volume ratios of *S. aureus*, *E. coli* and *C. albicans* ($V : V : V$) were gradually changed from 100 :

100 : 0, 91 : 91 : 18, 50 : 50 : 100 to 0 : 0 : 200, the corresponding number ratios of ($N$ : $N$ : $N$) were varied from 50 : 50 : 0, 50 : 50 : 10, 50 : 50 : 100 to 0 : 0 : 100, respectively. These bacteria/fungi mixtures were harvested by centrifugation (7100 rpm, 2 min). Similarly, the supernatant was removed, 1 mL of health urine was added to disperse the harvested pathogens. In this way, the hospital-acquired infection models were obtained. Next, the created urine samples were visually identified by the naked-eyes or under a fluorescence microscope. For the naked-eye visual approach, 10 μL of urine infection samples were transferred to 10 mL of LB and YPD medium, respectively, and then grown with a shaking speed of 220 rpm at 33 °C until the appearance of turbidity (about 5 – 8 h). The pathogens were centrifuged to harvest, resuspended with PBS, and then adjusted to a pathogen concentration of $OD_{600}$ = 1.0 or 2.0. Then the naked-eye identification operations were as same as that described in the experiment of naked-eye identification for pathogens. For visually identifying the urine samples by fluorescence microscope, 1 mL of created urine infection samples were first centrifuged to remove the supernatant urine, the remaining pathogen droplets were dispersed in 200 μL of PBS, then incubated with 10 μM of IQ-Cm in PBS solution for 10 min for imaging. The imaging operations were same as that in the experiment of pathogen staining and imaging. For monitoring the occurrence and evolution from the bacterial infection to fungal infection, the created urine samples with different mixed volume ratios of bacteria and fungi were treated in the same conditions as that of identifying the urine infection samples, and then imaged with the fluorescence microscope.

**Mold detection.** Four kinds of representative foods including persimmon, tomato, orange and bread were chosen as the tested samples. These samples were placed for about one week in the dark and damp environment. Apparently, persimmon was not moldy, the stem of tomato was rotten, and orange and bread got obviously moldy. The mold was collected from the circle range with the diameter of ~1 cm for detection. Among them, the white sugar icing part of persimmon and the bottom part of tomato after removing the rotten stem were chosen. The collected mold samples were dispersed in 1 mL of PBS solution, treated with 10 μM of IQ-Cm, and then incubated at room temperature for about 20-30 min. The

samples were observed under 365 nm UV irradiation and imaged with fluorescence microscope. The imaging operations were the same as that of *C. albicans* sample described in the pathogen staining and imaging experiment.

## Supplementary Figures and Tables

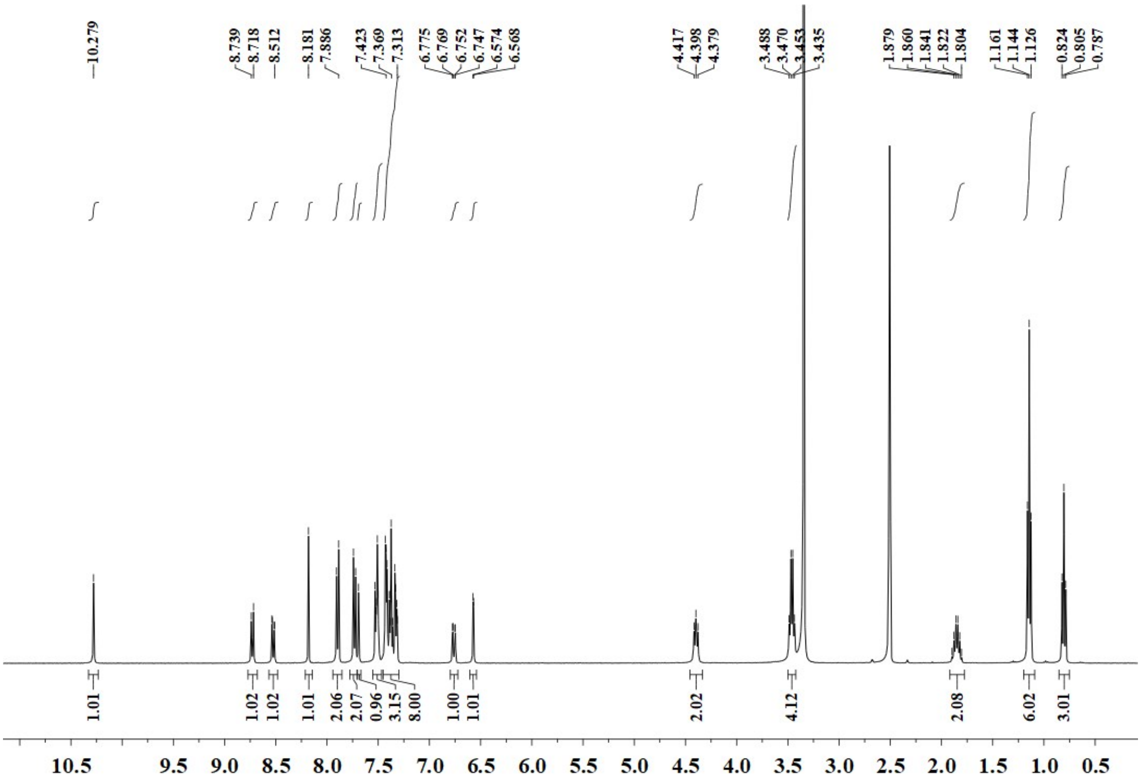

**Fig. S1** $^1$H NMR spectrum of IQ-Cm in $d_6$-DMSO.

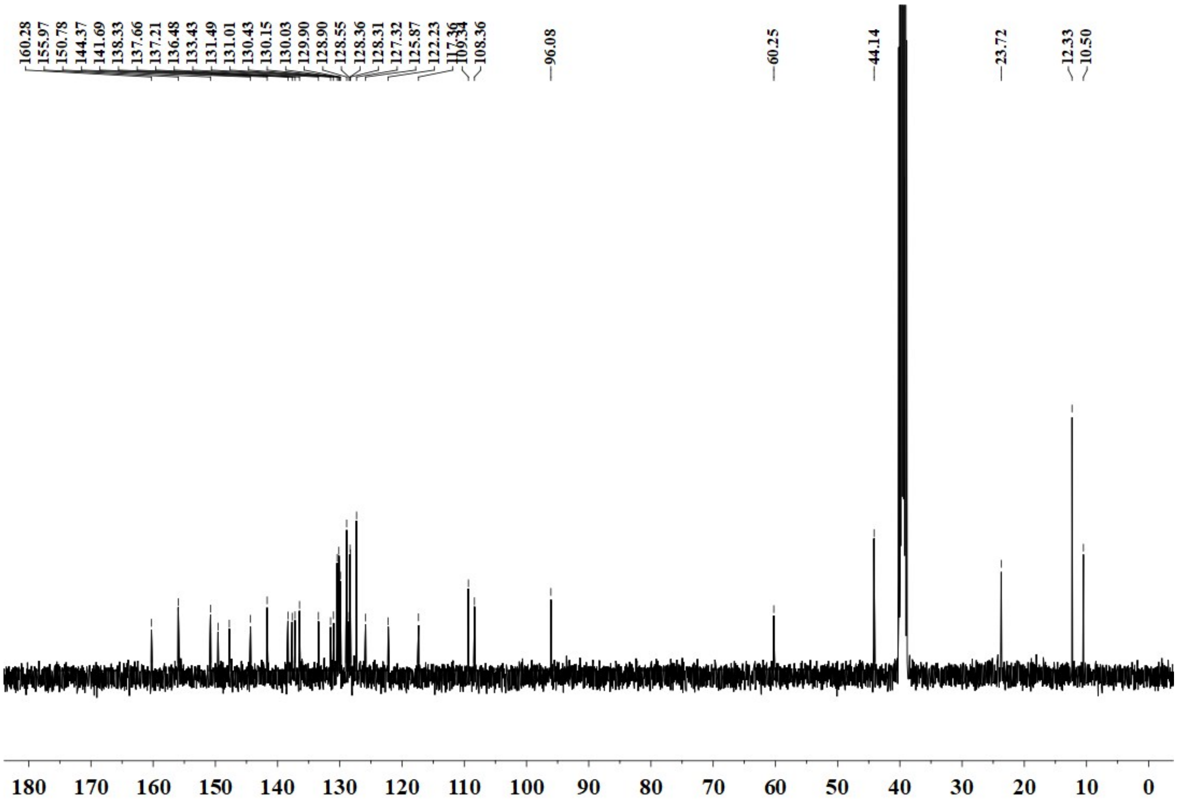


**Fig. S2** $^{13}C$ NMR spectrum of IQ-Cm in $d_6$-DMSO.

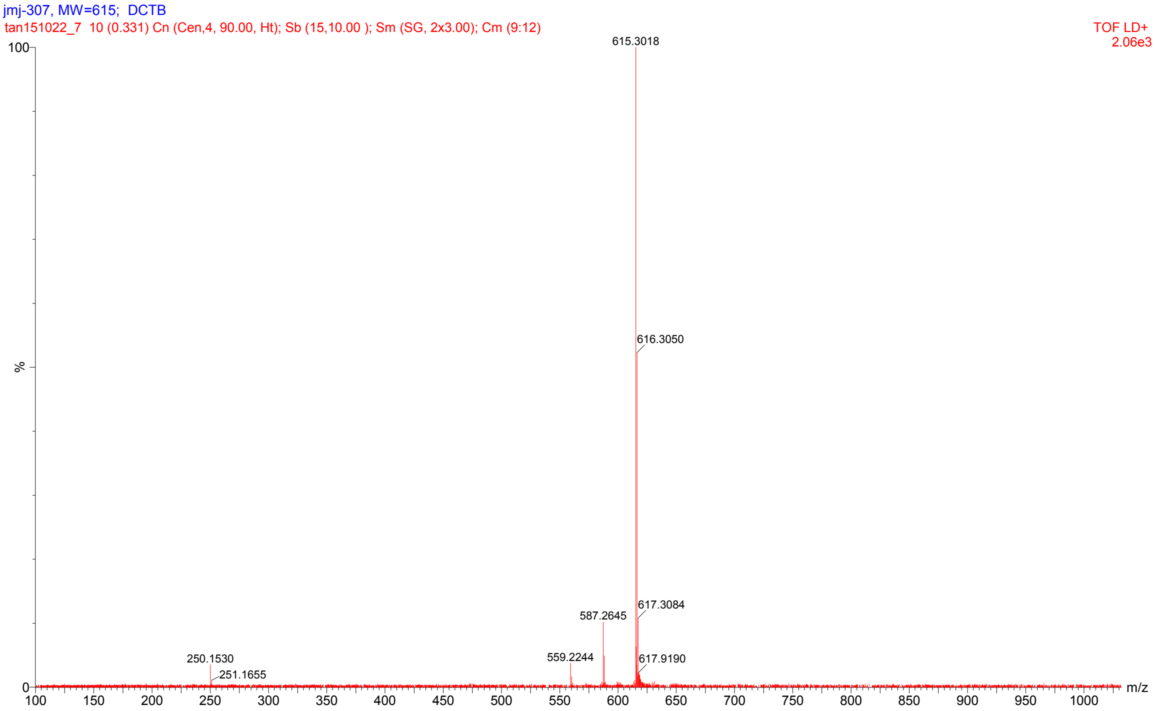


**Fig. S3** HRMS spectrum of IQ-Cm.

**Table S1** Crystal data and structure refinement for IQ-Cm.

| | | |
|---|---|---|
| Empirical formula | C43 H39 B F4 N2 O2 | |
| Formula weight | 702.57 | |
| Temperature | 293(2) K | |
| Wavelength | 0.71069 Å | |
| Crystal system | Monoclinic | |
| Space group | P $2_1$/c | |
| Unit cell dimensions | a = 14.5020(12) Å | □= 90°. |
| | b = 12.8660(9) Å | □= 106.828(5)°. |
| | c = 20.3200(19) Å | □ = 90°. |
| Volume | 3629.0(5) $Å^3$ | |
| Z | 4 | |
| Density (calculated) | 1.286 $Mg/m^3$ | |
| Absorption coefficient | 0.092 $mm^{-1}$ | |
| F(000) | 1472 | |
| Crystal size | 0.16 x 0.12 x 0.12 $mm^3$ | |
| Theta range for data collection | 2.206 to 25.061°. | |
| Index ranges | -17<=h<=17, -15<=k<=15, -24<=l<=24 | |
| Reflections collected | 36889 | |
| Independent reflections | 6272 [R(int) = 0.1746] | |
| Completeness to theta = 25.061° | 97.5 % | |
| Absorption correction | None | |
| Refinement method | Full-matrix least-squares on $F^2$ | |
| Data / restraints / parameters | 6272 / 208 / 508 | |
| Goodness-of-fit on $F^2$ | 1.156 | |
| Final R indices [I>2sigma(I)] | R1 = 0.0914, wR2 = 0.2067 | |
| R indices (all data) | R1 = 0.2050, wR2 = 0.2420 | |
| Extinction coefficient | n/a | |
| Largest diff. peak and hole | 0.636 and -0.276 e.$Å^{-3}$ | |

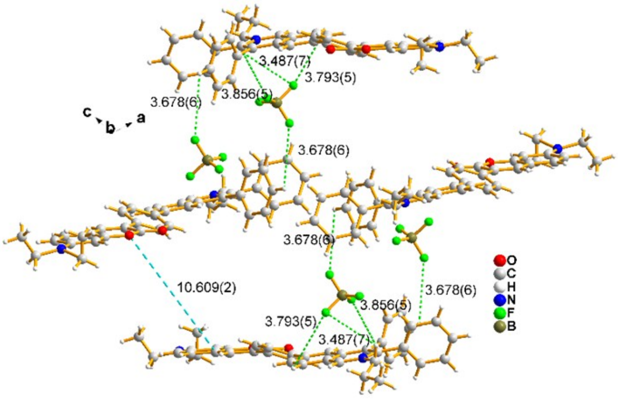


**Fig. S4** Intermolecular interactions in crystal structure. Hydrogen, carbon, nitrogen, oxygen, boron and fluorine are shown in white, gray, blue, red, breen and green, respectively.

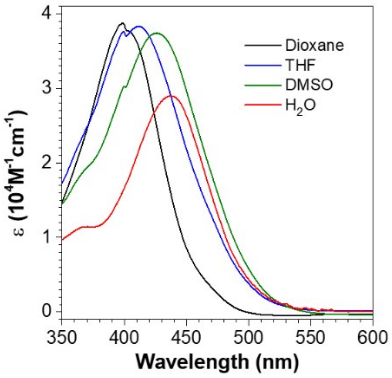


**Fig. S5** Absorption spectra of IQ-Cm in different solvents (Dioxane, THF, DMSO and $H_2O$ with 1% DMSO).

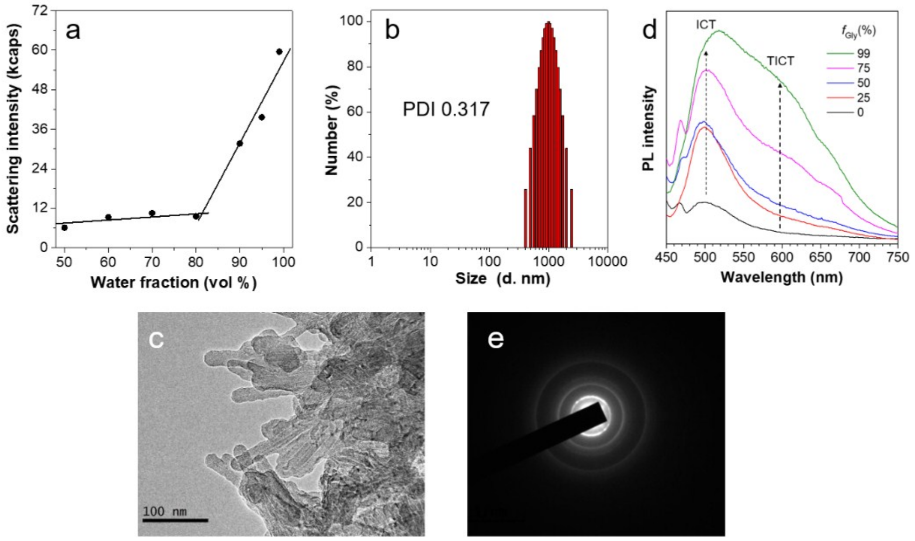


**Fig. S6** (a) Plot of scattering intensity of IQ-Cm versus water fraction in DMSO/$H_2O$ mixtures. (b) Size distribution, (c) TEM image and (e) transmission electron diffraction (TED) pattern of 20 μM of IQ-Cm in DMSO/$H_2O$ mixture with 98% water. (d) Fluorescence spectra of 10 μM of IQ-Cm in mixtures of ethylene glycol and glycerol with different glycerol fraction ($f_{Gly}$). Ex: 430 nm.

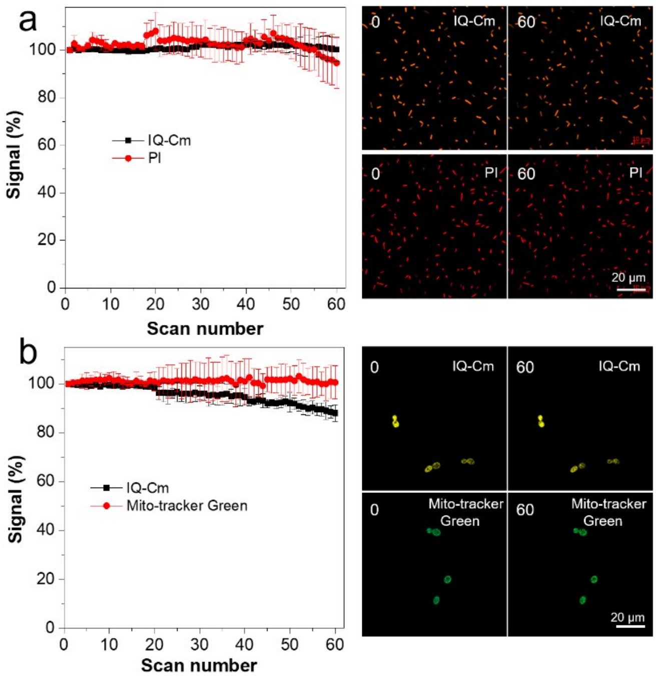


**Fig. S7** Photostability of IQ-Cm, PI and Mito-tracker Green. (a) Fluorescence signal change of dead *E. coli* (treated by 75% alcohol) stained with IQ-Cm and PI upon continuous scanning by laser light, respectively as well as their corresponding CLSM images stained with IQ-Cm (upper panel) and PI (lower panel) before and after 60 scans of laser irradiation. Concentration of IQ-Cm and PI: 10 μM. Imaging Conditions: Ex: 488 nm (0.5% laser intensity), Em: 500-700 nm for IQ-Cm and Em: 600-700 nm for PI. (b) Fluorescence signal change of *C. albicans* stained with IQ-Cm and Mito-tracker Green with the number of scans of laser irradiation, respectively as well as their corresponding CLSM images before and after 60 scans of laser irradiation. Concentration of IQ-Cm and Mito-tracker Green: 1.0 μM. Imaging Conditions: Ex: 488 nm (0.8% laser intensity), Em: 500-700 nm for IQ-Cm and Mito-tracker Green.

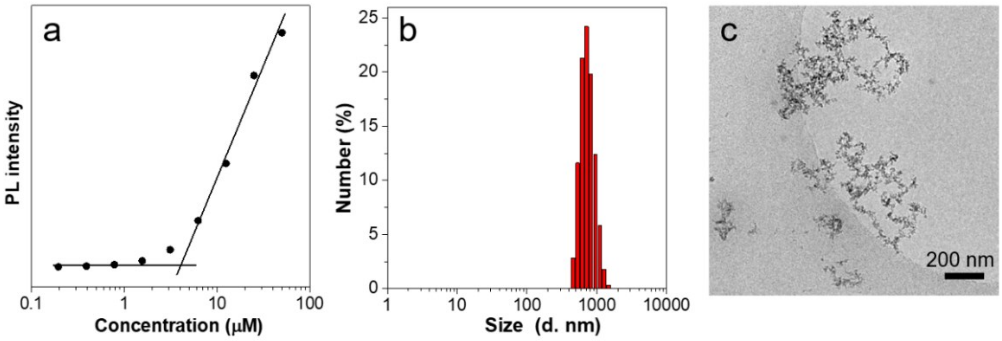


**Fig. S8** (a) Plot of fluorescence intensity at 650 nm of IQ-Cm versus the concentration in PBS solution, Ex: 450 nm (Based on the clear breakpoint, the critical aggregation concentration value of IQ-Cm was determined to be about 4 μM). (b) Size distribution and (c) cryo-TEM image of IQ-Cm at the concentration of 10 μM.

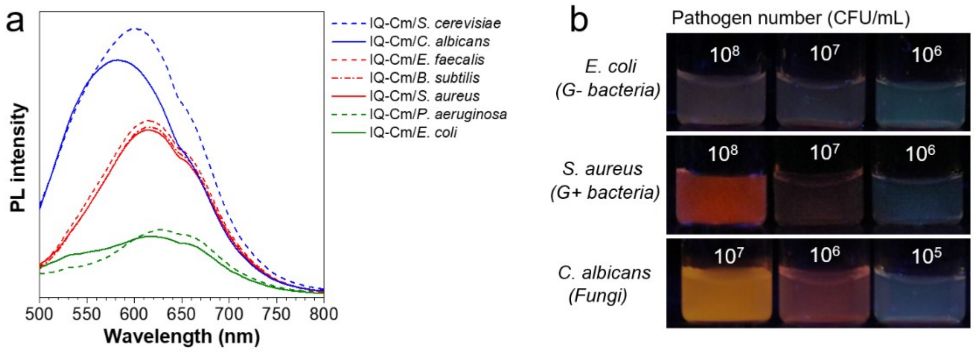


**Fig. S9** (a) Fluorescence spectra of 10 μM of IQ-Cm in PBS solution before and after adding pathogens, Ex: 450 nm. (b) The photographs of IQ-Cm taken under 365 nm UV irradiation before and after adding three pathogens (*E. coli*, *S. aureus*, *C. albicans)* with the different concentration.

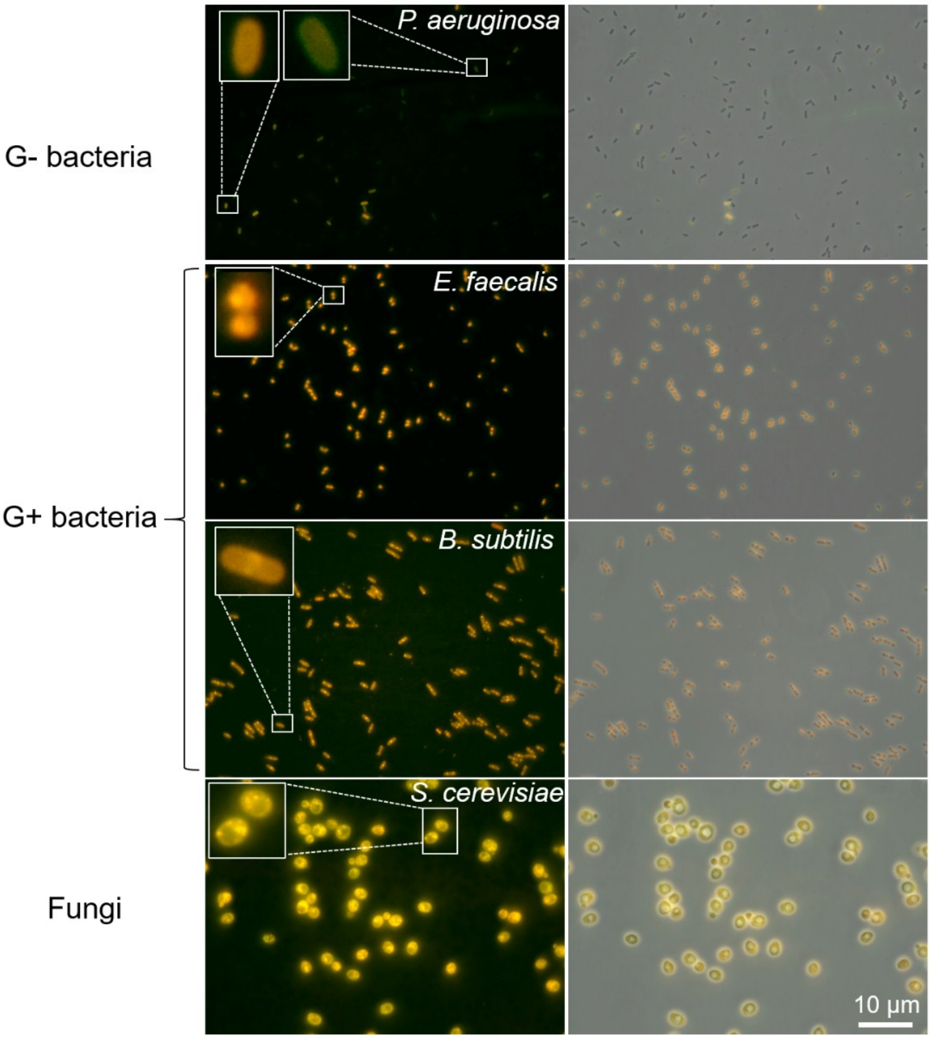


**Fig. S10** (a) Fluorescent field and merged images of *P. aeruginosa*, *E. faecalis*, *B. Subtilis* and *S. cerevisiae* incubated with 10 μM of IQ-Cm for 10 min. Imaging conditions: excitation filter = 460−490 nm, dichroic mirror = 505 nm, emission filter = 515 nm long pass.

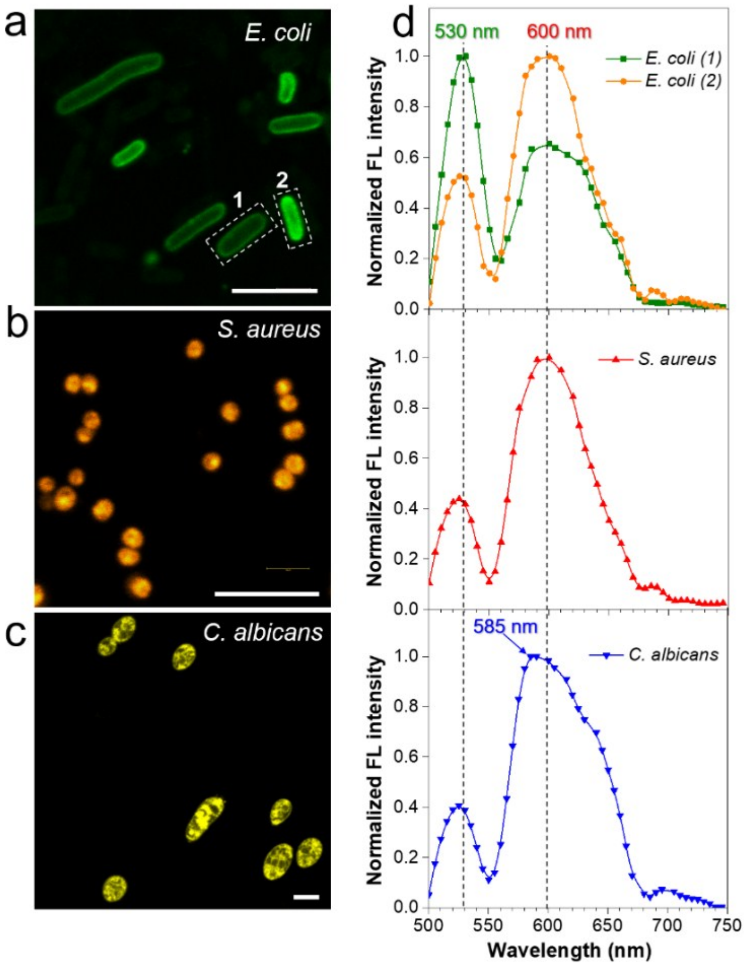


**Fig. S11** (a)-(c) CLSM images of *E. coli*, *S. aureus* and *C. albicans* incubated with 10 μM of IQ-Cm. Scale bar: 5 μm. (d) Fluorescence spectra of three pathogens stained with IQ-Cm collected in the wavelength scanning mode of CLSM with their corresponding CLSM images shown in (a)-(c). Ex: 488 nm.

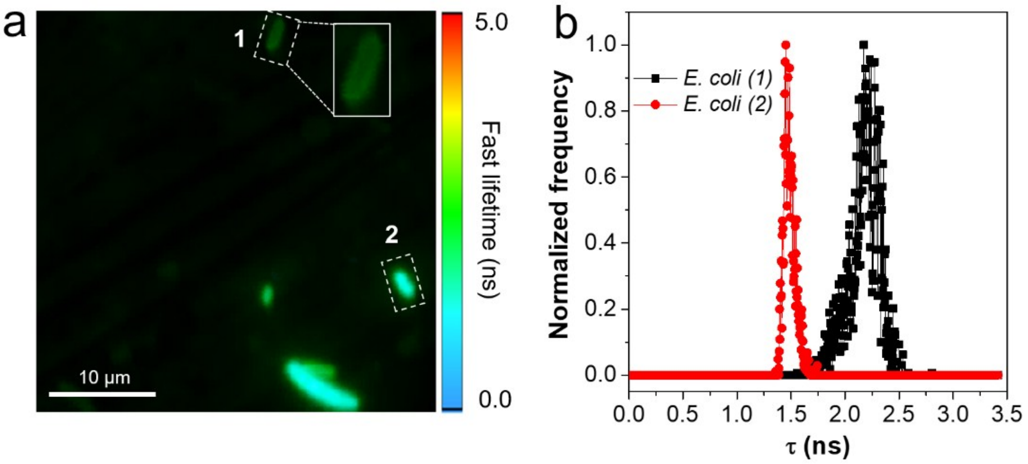


**Fig. S12** (a) Fluorescence lifetime imaging microscopy (FLIM) images of *E. coli* stained by 10 μM of IQ-Cm excited at 488 nm. (b) The fluorescence lifetime histogram of *E. coli* in the chosen range of (a).

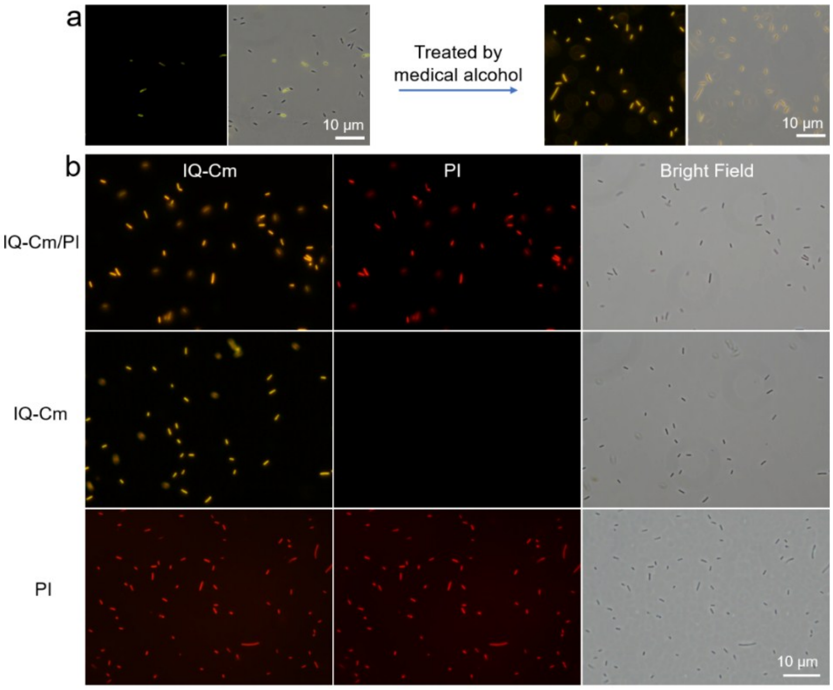


**Fig. S13** (a) Fluorescence microscopy images of *E. coli* before and after being treated by medical alcohol after stained with 10 μM of IQ-Cm in PBS solution. (b) Fluorescence microscopy images of *E. coli* treated by medical alcohol after being stained with IQ-Cm/PI, IQ-Cm and PI in PBS solution, respectively. [IQ-Cm] = 10 μM, [PI] = 5 μg/mL. Imaging conditions: excitation filter = 460−490 nm, dichroic mirror = 505 nm, emission filter = 515 nm long pass for IQ-Cm and excitation filter = 510−550 nm, dichroic mirror = 570 nm, emission filter = 590 nm long pass for PI.

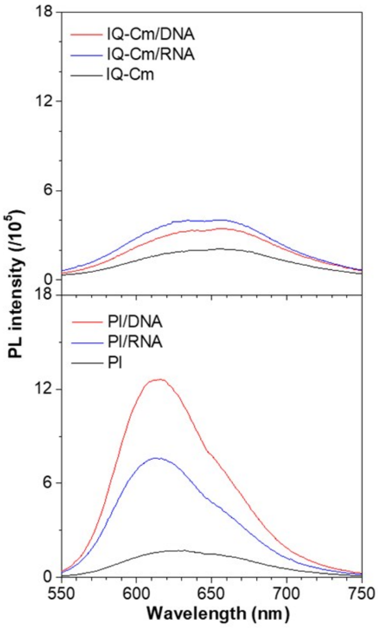


**Fig. S14** Fluorescence spectra of 10 μM of IQ-Cm and 5 μg/mL PI in PBS solution without and with 10 μg/mL of calf thymus DNA and RNA from torula yeast. $\lambda_{ex}$ = 450 nm for IQ-Cm and $\lambda_{ex}$ = 530 nm for PI.

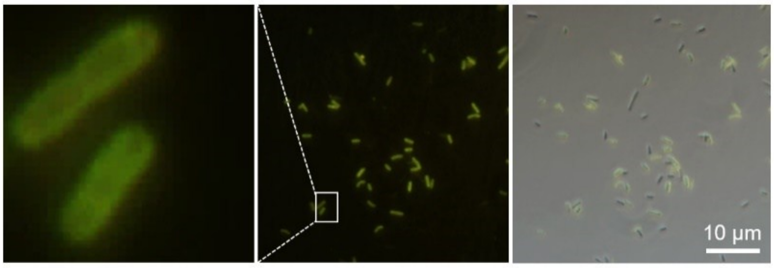


**Fig. S15** (a) Fluorescent field, merged images and enlarged fluorescence images of *E. coli* with disrupted outer membrane after incubating with 10 μM of IQ-Cm in PBS solution.

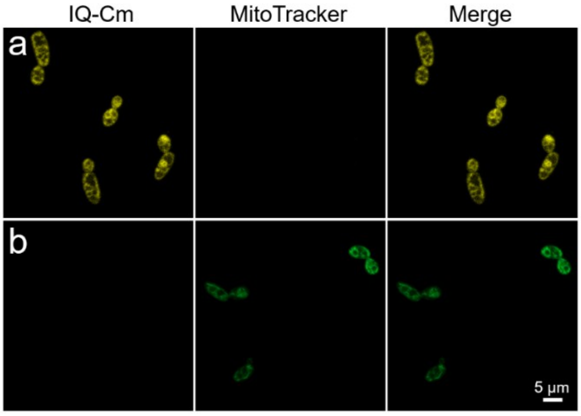


**Fig. S16** Confocal laser scanning microscope (CLSM) images of *C. albicans* stained with (a) 1 μM of IQ-Cm and (b) 100 nM of Mito-tracker Green in PBS solution for 10 min. Conditions: $\lambda_{ex}$ = 405 nm and $\lambda_{em}$ = 600-620 nm for IQ-Cm. $\lambda_{ex}$ = 488 nm and $\lambda_{em}$ = 500-520 nm for Mito-tracker Green.

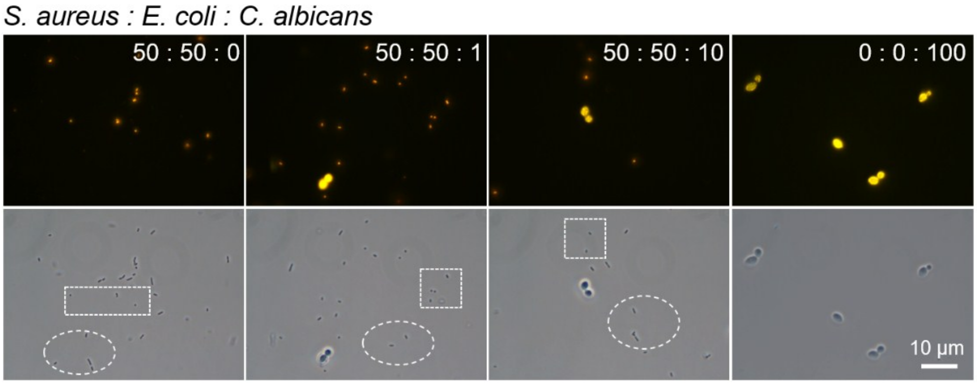


**Fig. S17** Fluorescence and bright field images of urine samples with different G+ bacteria/ G- bacteria/fungi ratio stained by IQ-Cm for 10 min. (G+ *S. aureus* and G- *E. coli* partly shown in the square and in the ellipse, respectively).

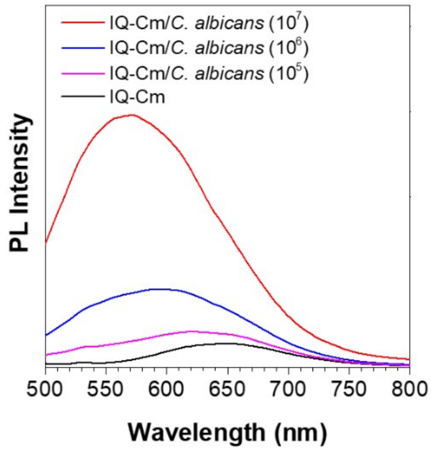


**Fig. S18** Fluorescence spectra of 10 μM of IQ-Cm before and after adding *C. albicans* with different concentration.

## References


1. T. Yu, J. Meng, P. Zhang, Y. Zhao, H. Zhang, D. Fan, L. Chen and Y. Qiu, *Spectrochimica Acta Part A: Mol. Biomol. Spectrosc.*, 2010, **75**, 1036-1042.
2. Z. Yang, Y. He, J.-H. Lee, N. Park, M. Suh, W.-S. Chae, J. Cao, X. Peng, H. Jung, C. Kang and J. S. Kim, *J. Am. Chem. Soc.*, 2013, **135**, 9181-9185.
3. J. Jayakumar, K. Parthasarathy and C. H. Cheng, *Angew. Chem. Int. Ed.*, 2012, **51**, 197-200.
4. E. Zhao, Y. Hong, S. Chen, C. W. Leung, C. Y. Chan, R. T. Kwok, J. W. Lam and B. Z. Tang, *Adv. Healthcare Mater.*, 2014, **3**, 88-96.
5. C. Zhou, D. Wang, M. Cao, Y. Chen, Z. Liu, C. Wu, H. Xu, S. Wang and Y. Wang, *ACS Appl. Mater. Interfaces*, 2016, **8**, 30811-30823.
6. R. M. Ribeiro, P. Rossi, H. G. C. Guidi and J. A. Pinotti, *Int. Urogynecol. J.*, 2002, **13**, 198-203.
